# Recent developments in anticancer kinase inhibitors based on the pyrazolo[3,4-*d*]pyrimidine scaffold

**DOI:** 10.1039/d0md00227e

**Published:** 2020-09-08

**Authors:** Daniel J. Baillache, Asier Unciti-Broceta

**Affiliations:** a Cancer Research UK Edinburgh Centre , Institute of Genetics and Molecular Medicine , University of Edinburgh , Crewe Road South , Edinburgh EH4 2XR , UK . Email: Asier.Unciti-Broceta@igmm.ed.ac.uk

## Abstract

Pyrazolo[3,4-*d*]pyrimidines have become of significant interest for the medicinal chemistry community as a privileged scaffold for the development of kinase inhibitors to treat a range of diseases, including cancer. This fused nitrogen-containing heterocycle is an isostere of the adenine ring of ATP, allowing the molecules to mimic hinge region binding interactions in kinase active sites. Similarities in kinase ATP sites can be exploited to direct the activity and selectivity of pyrazolo[3,4-*d*]pyrimidines to multiple oncogenic targets through focussed chemical modification. As a result, pharma and academic efforts have succeeded in progressing several pyrazolo[3,4-*d*]pyrimidines to clinical trials, including the BTK inhibitor ibrutinib, which has been approved for the treatment of several B-cell cancers. In this review, we examine the pyrazolo[3,4-*d*]pyrimidines currently in clinical trials for oncology patients, as well as those published in the literature during the last 5 years for different anticancer indications.

## Abstract 2

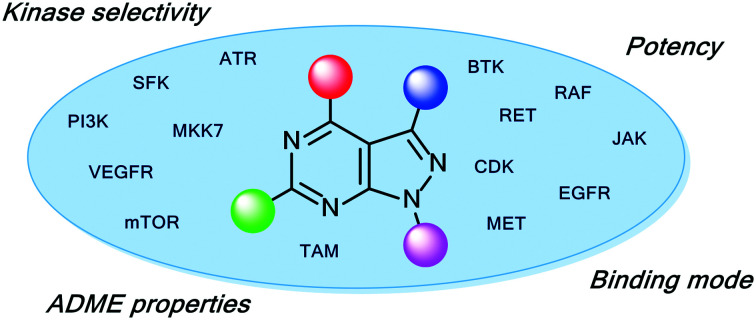
Pyrazolo[3,4-*d*]pyrimidines have become of significant interest for the medicinal chemistry community as a privileged scaffold for the development of kinase inhibitors to treat a range of diseases, including cancer.

## Introduction

Cancer is amongst the leading causes of death worldwide, accounting for approximately 10 million deaths each year.^1^,^2^ While some cancers are treatable and patients can benefit from high survival rates, several cancers have significantly lower survival rates, representing a significant unmet clinical need. Cancers characterised by high mortality that require more effective treatments include lung, brain, oesophageal and pancreatic cancers.^3^ The development of new treatments is challenging as cancer is a complex and heterogeneous disease, characterised by multiple mutational profiles underpinning its numerous common hallmarks.^4^ Self-stimulated proliferation, resistance to programmed cell death, limitless replicative potential and high levels of invasion are all common traits of cancer that can result from the overactivation of oncogenic pathways and/or the deactivation of tumour suppressor mechanisms.^5^

Protein kinases are enzymes responsible for the transfer of a phosphate group from ATP to a substrate. 518 different protein kinase genes have been identified to date.^6^ Based on the kinase substructure and the residues which they phosphorylate, the kinases can be further grouped into families and sub-families.^6^ Kinases play a critical role in many different cellular processes essential in the development and progression of disease, including cancer, inflammation, fibrosis, and neurological diseases.^6^,^7^ In cancer, mutations in kinases can trigger oncogenesis and are key contributors to cancer progression.^8^ As a result, kinase inhibitors have become a highly important class of oncodrugs in the last two decades.

Following the approval of the first kinase inhibitor in 2001, imatinib (Gleevec), a BCR-ABL inhibitor used to treat chronic myeloid leukaemia and acute lymphocytic leukaemia, over 50 kinase inhibitors have been approved for a variety of indications, from cancer to fibrosis and inflammation, amongst others.^9^,^10^ More than 45 of these inhibitors have been approved for the treatment of cancer, a number that grows quickly every year.^9^ Further to the approved inhibitors, over 200 small molecule kinase inhibitors are currently in clinical trials for many indications, though this figure is likely to be far higher.^11^ All but two approved inhibitors are administered orally, indicating that this class of inhibitors share structural characteristics and physicochemical properties.^9^ Most kinase inhibitors prevent the phosphorylation process, primarily through competition with the phosphate-donating ATP or the kinase substrate. Several studies have examined the possible binding modes for kinases inhibitors, with multiple modes being defined based upon how the inhibitor and kinase interact with eachother.^9^

**Fig. 1:**
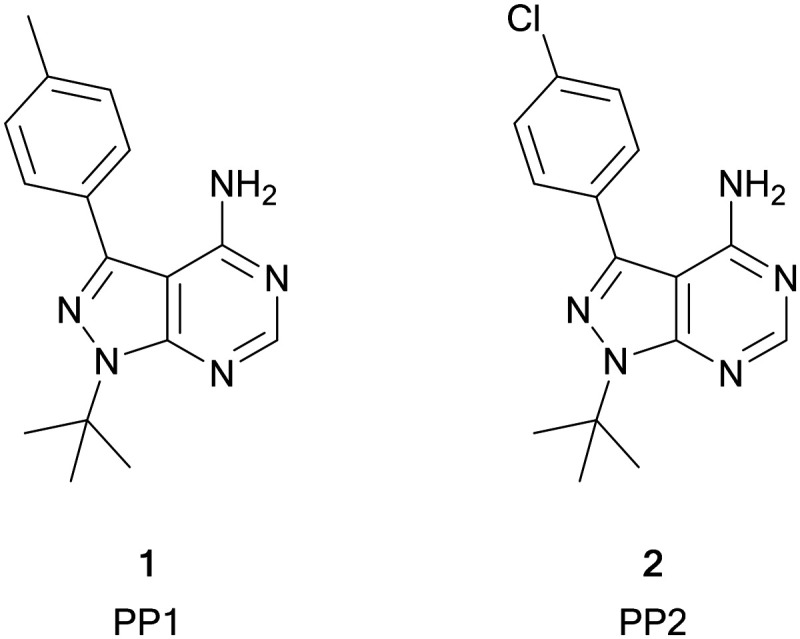
Structures of the first reported pyrazolo[3,4-*d*]pyrimidine kinase inhibitors **1** and **2**.

**Fig. 2:**
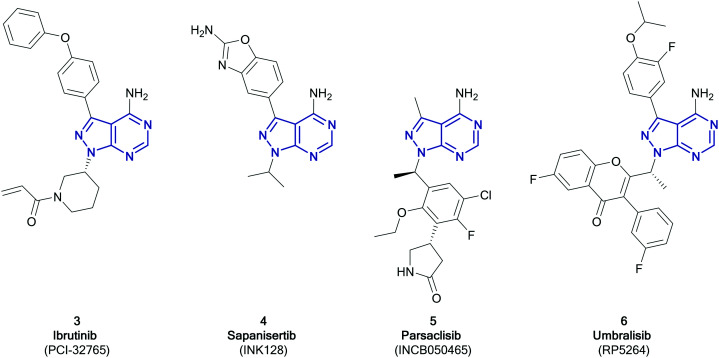
Compounds currently approved or undergoing clinical trials for cancer treatment that contain the pyrazolo[3,4-*d*]pyrimidine scaffold (highlighted in blue).

Among the variety of privileged heterocycles explored in the design of small molecule drugs, pyrazolopyrimidines are one of the most versatile.^12^,^13^ Comprised of a fused pyrazolo ring and pyrimidine ring, this scaffold is a bioisostere of adenine and can mimic key interactions of ATP with the hinge region of the kinase domain.^13^,^14^ This family of molecules can be further subdivided in various isomeric forms based on the configuration of the nitrogen atoms around the heterocycle. One of the most employed ones in drug discovery is the pyrazolo[3,4-*d*]pyrimidine bicycle, which has been shown to possess great potential as the basis of pharmacological agents for a range of indications, including antivirals, antimicrobials, antitumor agents, pesticides, CNS-agents, *etc.*^13^ To the best of our knowledge, the first pyrazolo[3,4-*d*]pyrimidine kinase inhibitors identified were PP1 (**1**) and PP2 (**2**), which were first discovered to act as kinase inhibitors of the SRC family of non-receptor tyrosine kinases in 1996 (Fig. 1).^15^ Years later, in 2013, ibrutinib (Imbruvica), **3** (Fig. 2), was approved by the FDA for the treatment of B-cell cancers.^16^ The discovery of **3** was unique for two reasons; firstly it was the first example of an irreversible kinase inhibitor to be approved, as the α,β-unsaturated carbonyl moiety is able to undergo a Michael addition with the thiol of cysteine residues in its target kinase BTK.^17^,^18^ Secondly it was the first kinase inhibitor to be approved possessing a pyrazolo[3,4-*d*]pyrimidine core. **3** is a potent and selective inhibitor of B-cell activation. B-Cells play important roles in the body's immune system, regulating immune responses by producing antibodies and using their surface receptors to bind to and trigger specific responses against antigens. BTK is a key component of the B-cell antigen receptor (BCR) signalling pathway.^16^,^18^ Disruption in B-cell activities can result in autoimmune diseases such as arthritis, and mutations in B-cells and their precursors can cause a range of cancers, such as leukaemia and lymphoma.^18^**3** was originally approved for chronic lymphocytic leukaemia (CLL), and later for mantle cell lymphoma (MCL) and Waldenström macroglobulinemia, with approvals for other lymphomas following. Since then, it has entered clinical trials for other indications, such as glioblastoma, breast, lung and pancreatic cancers,^19^,^20^ and it was approved for the treatment of graft *vs.* host disease in 2017.

## Pyrazolo[3,4-*d*]pyrimidines in clinical trials

While only **3** has so far been approved as a pyrazolo[3,4-*d*]pyrimidine containing kinase inhibitor, at present, a further three molecules are in clinical trials; parsaclisib, sapanisertib and umbralisib.^11^

Sapanisertib (otherwise known as INK128 or MLN1028, **4**, Fig. 2) is an experimental kinase inhibitor, currently in clinical trials for several cancer indications.^20^,^21^ **4** is an example of a mTOR inhibitor, inhibiting both the mTORC1 and 2 complexes. Mutations in mTOR signalling pathways are present in cancer cell growth and development. Prior to the development of mTOR inhibitors such as **4**, only rapalogs were known to inhibit the mTOR pathway by selectively inhibiting mTORC1, which results in a reduction of their anticancer efficacy due to compensatory activation of the mTORC2 complex. Dual mTORC1/2 inhibitors are proposed to be more effective.^22^,^23^ **4** is an ATP-competitive inhibitor, binding to the active site of mTOR with a *K*_i_ of 1.4 nM, while achieving high selectivity *versus* PI3Ks (*K*_i_ > 200 nM),^24^ although this selectivity profile has been put in doubt.^25^ This candidate is currently under investigation for an array of cancers, predominantly solid tumours. With phase I studies in glioblastoma, lung cancer, liver cancer and other solid tumours currently ongoing, it has already progressed to phase II studies in pancreatic, breast, lung cancers and leukaemia, administered both alone and in combination.^23^,^26^ Further studies have demonstrated efficacy in prostate cancer.^27^ The structure activity relationships of **4** have been further explored by chemists, with studies demonstrating that small structural changes can significantly alter its selectivity profile.^28^,^29^

Parsaclisib (otherwise known as INCB050465, **5**, Fig. 2) was first reported in 2014 by Incyte, being the product of a medicinal chemistry programme originating from the PI3Kδ inhibitor dezapelisib, which is currently undergoing phase II clinical trials for lymphomas.^20^,^30^,^31^ Structural modifications to dezapelisib, notably the alteration of the purine hinge-binder in favour of a pyrazolo[3,4-*d*]pyrimidine, yielded a series of highly potent PI3Kδ inhibitors, which will be discussed later in this article.^31^**5**, which possesses excellent selectivity for PI3Kδ over the other PI3K isoforms, showed the most promising pharmacological profile and was taken forward for further evaluation. The PI3Kδ isoform plays a central role in the signalling network that controls B-cell growth, and it has been shown that mutations in this kinase are responsible for the formation of B-cell cancers.^32^**5** has entered clinical trials in a range of indications, such as lymphoma, breast cancer, and other solid tumours, with some success progressing through phases, particularly against lymphomas.^20^

Umbralisib (otherwise known as RP5264, **6**, Fig. 2) is an investigational dual PI3Kδ/CK1 inhibitor, first disclosed in 2014, currently in phase III clinical trials for the treatment of lymphomas, particularly for marginal zone lymphoma, which has no current FDA-approved treatment.^20^,^33^ **6** has been shown to be an effective treatment of B-cell cancers, due to its PI3Kδ activity. **6** also inhibits CK1, which is responsible for mRNA translation, as well as having greater selectivity for the PI3Kδ-isoform than other inhibitors.^34^,^35^ Studies comparing the effect of **6** relative to idelalisib (the first PI3Kδ inhibitor to be approved, which is structurally similar to **6**), attribute its greater efficacy against more aggressive lymphomas to the dual inhibition of CK1 in conjunction with PI3Kδ, and the subsequent reduction in the levels of the c-MYC transcription factor.^36^ Following a successful phase I study in patients with leukaemia and lymphoma, **6** passed through a phase II study to phase III, where it has been awarded breakthrough therapy status by the FDA.^20^,^35^,^37^

## Pyrazolo[3,4-*d*]pyrimidines in preclinical development

Pyrazolo[3,4-*d*]pyrimidines continue to be of great interest in drug discovery programmes, especially those in cancer drug discovery. In the next sections, we discuss recent publications of anticancer kinase inhibitors featuring this scaffold, grouped by the kinases which they inhibit.

### ATR

Ataxia telangiectasia and rad-3 related protein (ATR) is a member of the phosphatidylinositol-3-kinase-related protein kinase (PIKK) family of serine/threonine protein kinases. ATR senses DNA damage and triggers repair through cell cycle arrest of damaged cells.^38^ Despite several ATR-inhibitors currently undergoing clinical trials, none yet are approved. Current compounds, such as ceralasertib (AZD6738) and BAY1895344, are undergoing evaluation for the treatment of leukaemia and lymphoma.^39^,^40^

**Fig. 3:**
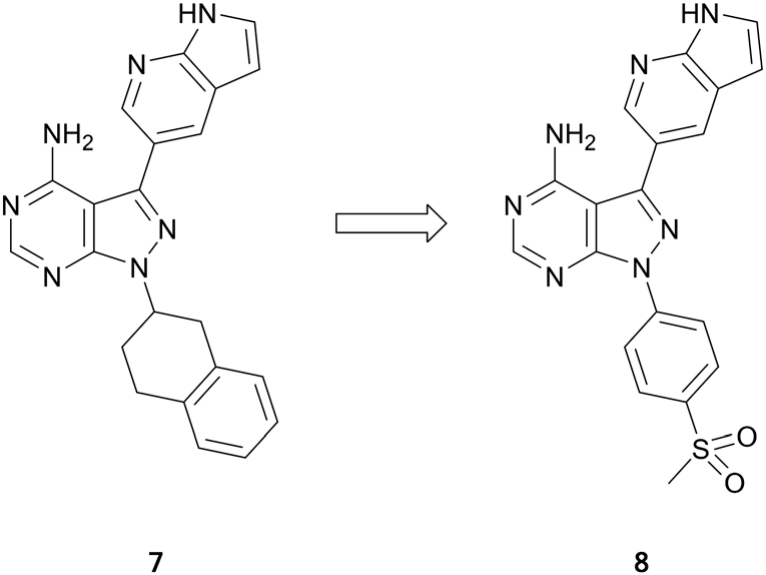
Optimisation of pyrazolo[3,4-*d*]pyrimidines for the development of ATR kinase inhibitors.

The PIKK family has a degree of homology with the PI3K family and these molecules can be used as a means for guiding ATR inhibitor design.^41^ A library of PI3K and BTK inhibitors was used by Ramachandran *et al.* to find ATR hits through an screening campaign. Pyrazolo[3,4-*d*]pyrimidine **7** was identified as of interest (Fig. 3), as in addition to being the most potent ATR inhibitor, it also demonstrated selectivity *versus* BTK and PI3Kδ. Small focussed libraries were designed to explore structure activity relationships (SAR) around the ring. Through several iterations of synthesis and screening, **8** was identified with a high potency against ATR (IC_50_ = 66.0 nM), a 3-fold improvement on the initial hit (Fig. 3). The selectivity of **8** with respect to 394 other kinases was determined. At 1 μM, **8** inhibited 23 kinases at levels greater than 70%, with minimal activity against PI3Ks and BTK, and activity primarily against other tyrosine kinases.^42^

### BTK

Bruton's tyrosine kinase (BTK) is a key regulator in the B-cell receptor (BCR) signalling pathway. BTK overexpression is frequently observed in B-cell cancers such as lymphomas and leukaemia.^43^,^44^ The BTK active site contains an exposed cysteine residue which makes it possible for covalent inhibition of the kinase.^45^,^46^ The approval of **3** triggered numerous medicinal chemistry programmes aiming to develop further BTK inhibitors as well as inhibitors of other kinases containing conserved cysteine residues.^45^,^47^ While **3** is currently a highly successful treatment, it is only moderately selective *versus* other kinases and has some off-target toxicity, which is a result of the key cysteine residue being conserved in other kinases, such as EGFR and ITK.

The first of these ‘second-generation’ BTK inhibitors was acalabrutinib, which modified the structure of both the C3- and N1-positions of **3** to improve BTK potency and selectivity.^48^ The structures of the BTK inhibitors developed over subsequent years frequently conserve the pyrazolo[3,4-*d*]pyrimidine scaffold central to **3**. Some studies have focussed on exploring the properties of the Michael acceptor, through modifications to the α,β-unsaturated carbonyl moiety, while other studies have focussed on the ether-linked phenyl rings in the northern region, which lie near the DFG motif. The pyrazolo[3,4-*d*]pyrimidine scaffold has also been subject to alteration, such as conversion to other pyrazolopyrimidine isomers or heteroaromatic ring systems. However, these will not be covered here.

**Fig. 4:**
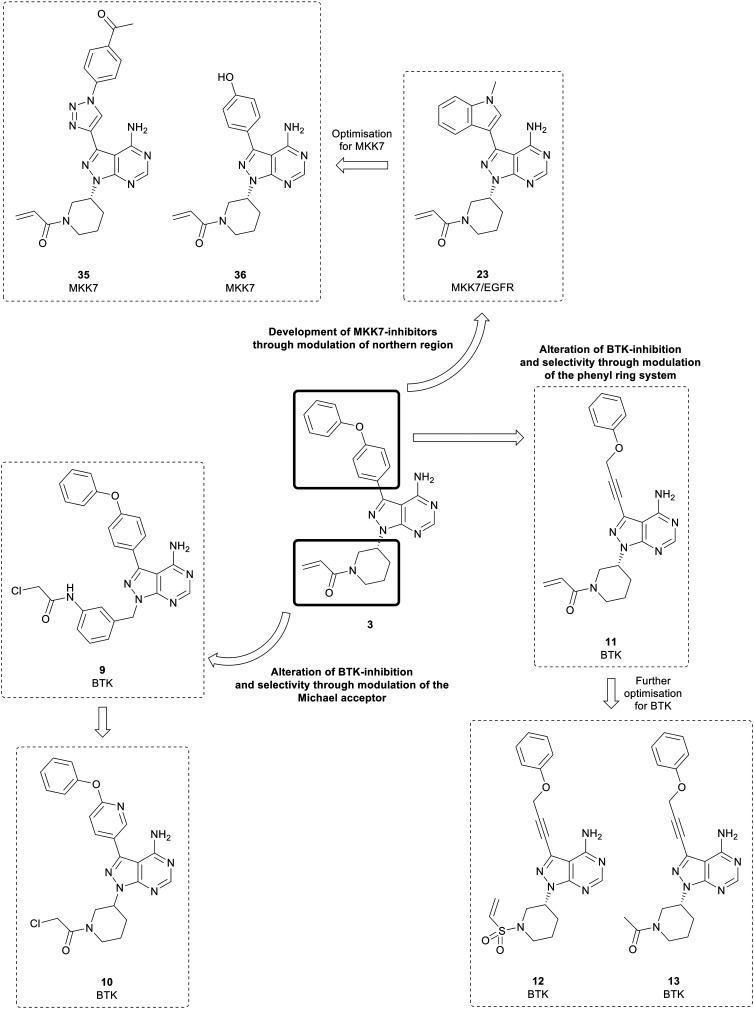
Pyrazolo[3,4-*d*]pyrimidine inhibitors of BTK, MKK7 and EGFR derived from the first-in-class covalent BTK inhibitor **3**.

Analysis of pyrazolo[3,4-*d*]pyrimidine BTK inhibitors identifies some common characteristics; a northern region at the C3-position of the central core that exploits a hydrophobic pocket, the central core itself, and the Michael acceptor warhead, which is attached to the core at the N1-position by a linker.^49^ Several studies have focussed on the warhead and have swapped the α,β-unsaturated carbonyl moiety in favour of more reactive groups such as chloroacetyl groups (Fig. 4). In one such study, the piperidine linked warhead of **3** was changed for derivatives of the benzyl-linked warhead found in the BTK inhibitor spebrutinib, yielding lead compound **9**. This compound maintained BTK-potency relative to **3**, and kinome profiling demonstrated a greater selectivity for BTK over a range of kinases, including EGFR.^49^ Optimisation of **9** sought to modify the phenyl rings in the C3-position of the molecule through the addition of pyridine rings in the linker to improve binding. Although most of the new derivatives were weakly potent for BTK, **10**, which incorporated a chloroacetyl warhead, demonstrated a low-nanomolar potency against BTK, with improved anticancer activity in a Mantle cell lymphoma cell line and improved selectivity over other kinases.^50^

Further studies have examined the groups in the C3-position, retaining the core and warhead key for BTK activity. π-Stacking interactions in the distal phenyl ring were explored by changing the linkers between the core and the phenyl ring (Fig. 4). **11**, with the phenyl linked to the core by an alkynyl ether, showed high potency against BTK comparable to **3**, with an IC_50_ value of 7.95 nM, whilst demonstrating a better physicochemical profile than the approved drug.^51^**11** was further optimised through the design and synthesis of 15 molecules containing alterations to the warhead in the N1-position. One of the derivatives synthesised, vinyl sulphonamide **12**, demonstrated an improved BTK IC_50_ than **3** at 4.2 nM. Despite this, upon evaluation in two B-cell leukaemia lines, **12** did not show effective antiproliferative activity. In contrast, another potent BTK-active molecule from the series containing an acetamide **13** (IC_50_ = 11.1 nM), exhibited a low micromolar cytotoxicity against B-cell cancer cell-lines.^52^ This is further proof of the limitation of ranking compounds' potencies only by biochemical assays against isolated proteins, as it overlooks the capacity of the compounds to cross biological barriers.

**Fig. 5:**
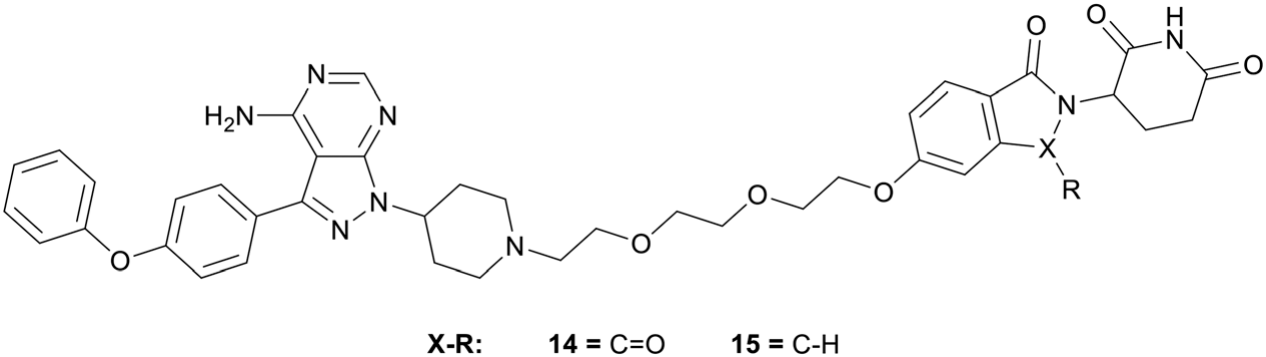
Design of PROTACs for the targeted degradation of BTK.

Proteolysis targeting chimeras (PROTACs) have emerged as a technology for selectively inhibiting a target by triggering proteasomal degradation of proteins after E3-ligase mediated ubiquitination.^53^,^54^ The PROTAC approach has been of specific interest in cases of chronic lymphocytic leukaemia, where drug resistance arises because of mutation of the active cysteine residue to a serine residue preventing covalent inhibition.^55^ To reduce off-target toxicity and to overcome drug resistance, **3** has been used to develop a BTK targeting PROTAC (Fig. 5). BTK PROTACs have been developed by linking an E3-ligase cereblon ligand to the pyrazolo[3,4-*d*]pyrimidine core with a polyethylene glycol linker, attached at the piperidine ring in the N1-position, replacing the covalent warhead. An initial PROTAC developed, MT802 (**14**), degrades BTK in cells at low nanomolar concentrations *in vitro*. However, high clearance and a resultant low half-life in mice discarded further preclinical development.^55^ Efforts have been made to improve these properties, while retaining the BTK-degradation properties, through the modification to the cereblon ligand, by removal of one of the carbonyl groups and the linking group to the glycol chain, yielding PROTAC SJF620 (**15**). **15** maintained a similar level of BTK degradation, and possessed a low clearance and a longer half-life.^56^

### CDK

The cyclin dependent kinases (CDKs) are a family of serine/threonine kinases that are cell cycle regulators. Only 5 out of the 12 CDK isoforms are directly involved in the control of the cell cycle. In cancer, dysregulation of the cell cycle results in rapid and uncontrolled proliferation of cancer cells, as well as resistance to treatment, making it an attractive target for anticancer agents.^57^,^58^ First generation CDK inhibitors, such as roscotovine and flavopiridol, were generally pan-CDK inhibitors, and CDK2 was a common target across the class. Given this lack of selectivity over non-oncogenic CDK isoforms, few of these inhibitors progressed further in clinical trials due to mild toxicity and poor efficacy.^57^ Second generation CDK inhibitors have also been developed, and are generally more selective. Three dual CDK4/6 inhibitors, palbociclib, ribociclib and abemaciclib, have been approved for the treatment of breast cancer.^58^

**Fig. 6:**
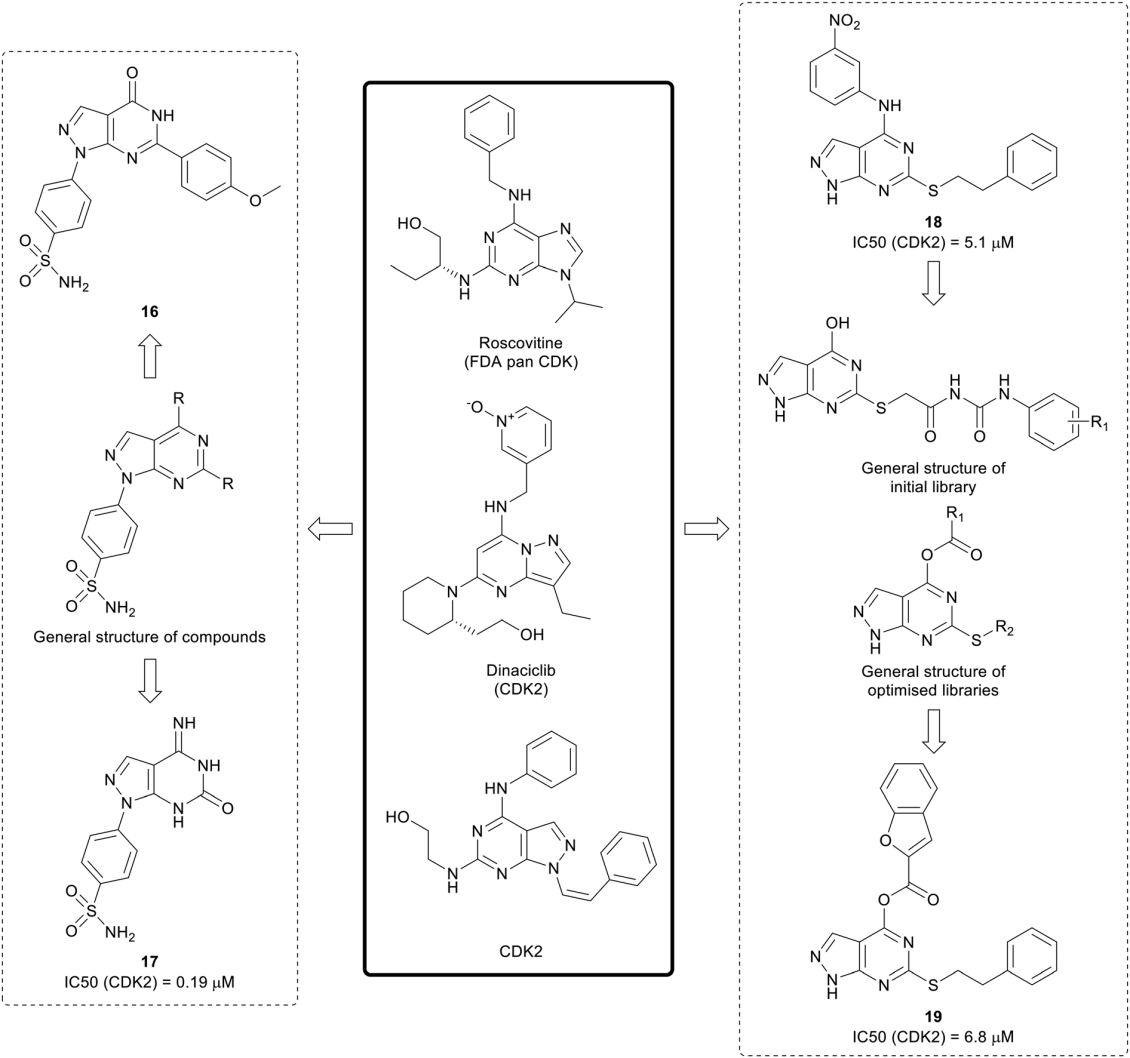
Development of CDK inhibitors from existing pyrazolopyrimidine and purine CDK inhibitors.

Existing suboptimal inhibitors such as roscovitine, led to the design of further inhibitors, such as dinaciclib, which contains a pyrazolo[1,5-*a*]pyrimidine, an isostere of the pyrazolo[3,4-*d*]pyrimidine scaffold.^59^,^60^ Using dinaciclib as a starting point, Hassan *et al.* designed a library of pyrazolo[3,4-*d*]pyrimidines that incorporated benzene sulphonamides in the N1-position (Fig. 6). Molecules containing varied substitutions at the C4- and C6-positions were synthesised, and screened in two cancer cell lines. The two most promising molecules **16** and **17** were shown to induce apoptosis. The most potent CDK2 inhibitor was **17** with an IC_50_ of 0.19 μM.^61^

Further research by Cherukpalli *et al.* has sought to exploit the pyrazolo[3,4-*d*]pyrimidine scaffold to generate CDK2 inhibitors. Based on existing pyrazolo[3,4-*d*]pyrimidines that have been developed as kinase inhibitors, such as the series of SRC family kinase inhibitors generated by Schenone *et al.*, a series of 4,6-disubstituted pyrazolo[3,4-*d*]pyrimidines were synthesised through incorporation of amine linked aryl groups in the C4-position and aliphatic or aromatic groups in the C6-position (Fig. 6). The molecules were screened against ABL, CDK2 and two cancer cell lines. A thiophenethyl in the C6-position was found to be essential for anticancer potency and the substitution pattern in the C4-aryl moiety was important for CDK2 inhibition. **18** showed the greatest CDK2 inhibition (IC_50_ = 5.1 μM) and it showed low-micromolar anticancer activity.^62^ The initial pyrazolo[3,4-*d*]pyrimidine SAR generated was taken forward to design further compounds, with a series optimised from an initial set of phenyl carbamoyl acetamide derivatives with a thioether linkage. Through modification and derivatisation of the C4- and C6-positions (Fig. 6), molecules were synthesised, and screened for *in vitro* enzymatic and antiproliferative activity. Several leads were identified and, of these, **19** demonstrated the best CDK2 inhibition (IC_50_ = 6.8 μM) and low-micromolar potency against two cancer cell lines.^63^

### cMET

**Fig. 7:**
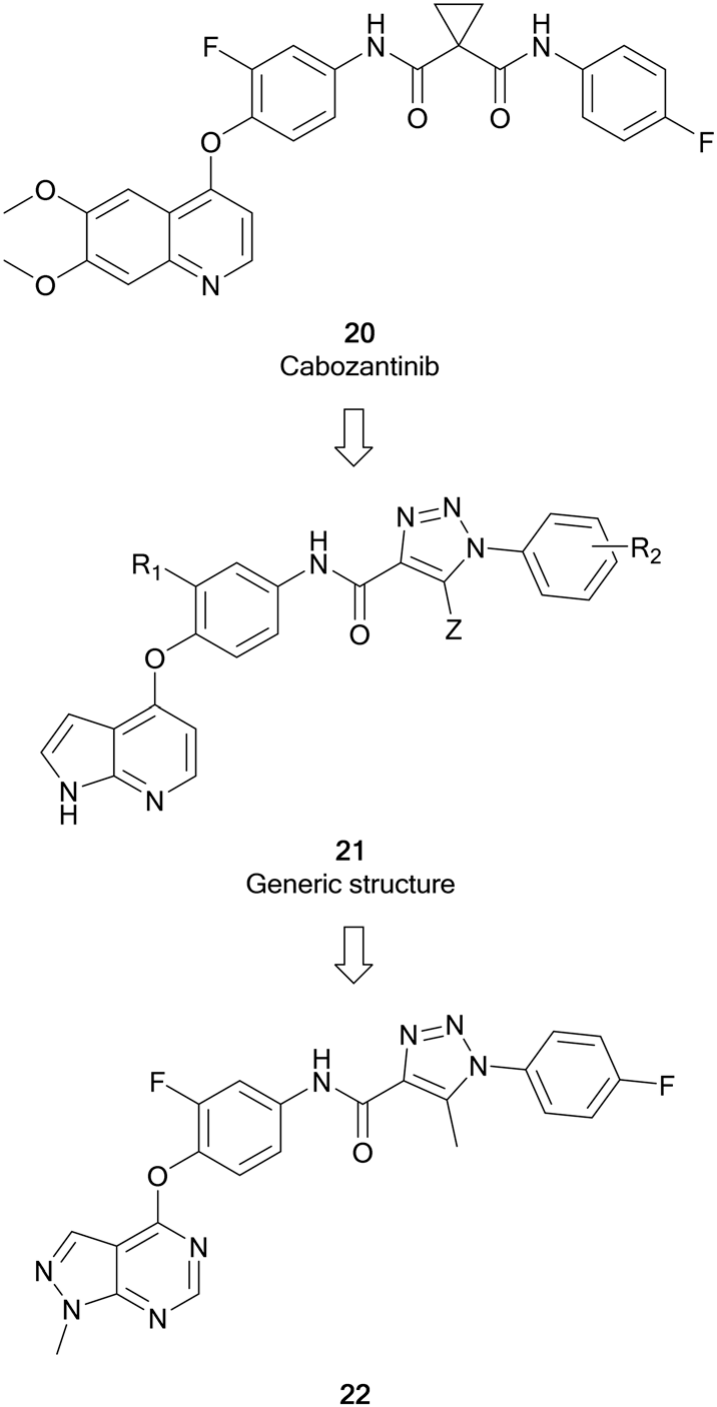
Development of c-MET inhibitors from **20**.

cMET is a member of the MET family of receptor tyrosine kinases. cMET is a transmembrane receptor expressed on the surface of cells that binds the hepatocyte growth factor (HGF) ligand. While under normal conditions mediates the wound healing response, abnormal cMET activation can promote the development and progression of multiple cancers.^64^ The HGF/cMET signalling cascade can stimulate downstream pathways implicated in cancer progression, such as PI3K/AKT, JAK/STAT, RAS/MAPK and SRC, which are responsible for regulating proliferation, invasion, metastasis and other hallmarks of cancer. cMET is frequently involved in the development of drug resistance against these pathways, associating it with poor prognosis and lower survival.^64^,^65^ Cabozantinib, **20**, was the first example of a FDA approved cMET inhibitor (Fig. 7). As a multi-targeting tyrosine kinase inhibitor, **20** also inhibits VEGFR2 and other kinases such as RET and AXL, and has been primarily used for the treatment of renal cell carcinoma and liver cancers.^66^ Further generations of cMET inhibitor have been developed, with capmatinib being the first selective cMET tyrosine kinase inhibitor approved, for resistant non-small cell lung cancer.^65^

Work by Wang *et al.* initially developed a series of pyrrolo[2,3-*b*]pyridine derivatives from **20** (Fig. 7). These molecules (**21**) maintained a key 5-atom linker between the pyrrolopyridine core and the distal phenyl ring. Generic structure, **21**, was optimised through replacement of one of the amine components of the urea linker for a triazole moiety. Further optimisation of **21** converted the pyrrolopyridine to a pyrazolo[3,4-*d*]pyrimidine bearing a triazole moiety with varied substitutions of the terminal ring. The library of molecules was screened against breast, liver and lung cancer cells to determine anticancer effect as well as evaluating the cMET inhibition. **22** was shown to be the most active of the pyrazolopyrimidine series with EC_50_ values of 10 μM or less, demonstrating antiproliferative effect in cancer cells. Evaluation of the cMET IC_50_ in biochemical assays showed that **22** possessed moderate potency with a low micromolar IC_50_.^67^

### EGFR

Epidermal growth factor receptor (EGFR) is a family of transmembrane receptor tyrosine kinases comprising of EGFR/ErbB1, HER2/ErbB2, HER3/ErbB3 and HER4/ErbB4. The EGFR family are major contributors to complex signalling cascades that control cell growth, differentiation, migration, and survival.^68^ Mutations and aberrations in EGFR activity play an important role in many malignancies, including breast, lung, and oesophageal cancers, amongst others, making EGFR an attractive target for intervention.^69^,^70^ Gefitinib, first characterised in 1996, was the first EGFR inhibitor to be approved by the FDA, for the treatment of NSCLC, in 2003. Further EGFR inhibitors, such as erlotinib, an anticancer drug used for breast and pancreatic cancers, and lapatinib followed soon after.^68^,^69^

Engel *et al.* used **1** and **3** as starting points for EGFR inhibitor design. A structure-based approach was used to design a small focussed library of inhibitors which explored the C3-position that extends towards a methionine residue in the active site. A key component within the structure of the most active inhibitors was the inclusion of a piperidine-linked Michael acceptor in the N1-position, which facilitates covalent binding to a conserved cysteine residue present in the active site of EGFR, much like that of BTK. Several molecules, of which the lead molecule was **23** (Fig. 4), were highly potent in a set of cancer cell lines and presented nanomolar potency against EGFR in biochemical assays. Further analysis of the physicochemical properties *in vitro* and *in vivo* demonstrated that **23** possessed the best overall profile, displaying a high kinetic solubility as well as an excellent permeability.^71^

**Fig. 8:**
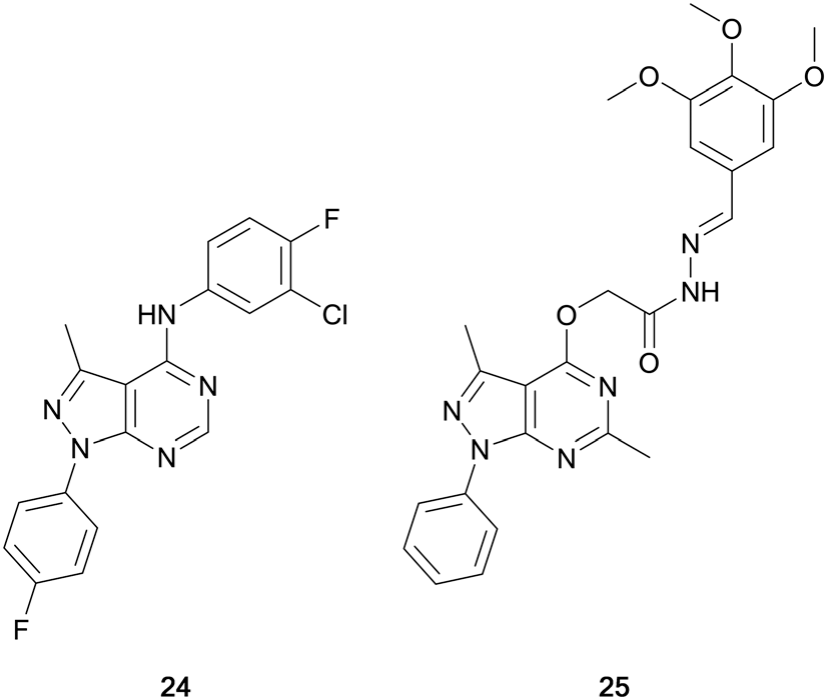
EGFR inhibitors developed through bioisosteric replacement of the quinazoline moiety of approved EGFR inhibitors erlotinib and lapatinib.

Maher *et al.* designed a series of dual EGFR/ErbB2 inhibitors through the isosteric replacement of the quinazoline core of lapatinib and erlotinib, with a pyrazolo[3,4-*d*]pyrimidine core. The substitutions around this core primarily focussed on the incorporation of a library of substituted arenes in the C4-position linked by a variety of different nitrogen-containing moieties. The 4-fluorobenzene moiety in the N1-position was maintained in the library. The compounds synthesised were screened against a panel of 60 cancer cell lines for percentage growth inhibition, and of the compounds screened, compound **24** showed moderate anticancer activity across the panel (Fig. 8). In several cell lines **24** demonstrated greater potency than the control, erlotinib. Further evaluation demonstrated sub-micromolar potency against the EGFR and HER2 kinases (IC_50_ = 186 and 254 nM, respectively), demonstrating its retention of dual inhibition, as well as exerting its antiproliferative activity by inducing cell cycle arrest.^72^

Abdelgawad *et al.* designed a series of ATP-competitive inhibitors based upon a series of successful anilinoquinazoline-containing compounds that reached and succeed in clinical trials, such as erlotinib and gefitinib. These molecules incorporated this moiety into the pyrazolo[3,4-*d*]pyrimidine scaffold, creating libraries which focussed predominantly on the substitutions to the distal aromatic ring. The compound library was screened in three cancer cell lines to evaluate their anticancer effect, with several molecules presenting low-micromolar potencies. Lead molecules were assayed against a panel of growth factor receptor kinases, and lead molecule **25** (Fig. 8) was identified as a selective EGFR inhibitor with low micromolar IC_50_ (4.18 μM).^73^

### JAK

The Janus kinases (JAK1, JAK2, JAK3 and TYK2) are a family of intracellular tyrosine kinases that regulates cell proliferation, differentiation, survival, and embryological processes.^74^ Oncogenic mutations can result in activation of the JAK–STAT pathway, contributing to the progression of several cancers, including haematological malignancies and some solid tumours.^75^ The JAK active site contains a conserved cysteine residue, like that of BTK and EGFR, and therefore covalent inhibition of this kinase is possible. JAK inhibitors with a range of selectivity profiles have been described, with seven inhibitors currently approved for clinical use and several others in clinical trials. Several of these inhibitors are indicated for the treatment of various cancers. Ruxolitinib, a JAK1/2 inhibitor, was approved for the treatment of myelofibrosis – a rare form of bone marrow cancer – in 2011 and is currently being investigated for the treatment of further B- and T-cell cancers. A second JAK2 inhibitor for the treatment of myelofibrosis, fedratinib, was approved in 2019.

**Fig. 9:**
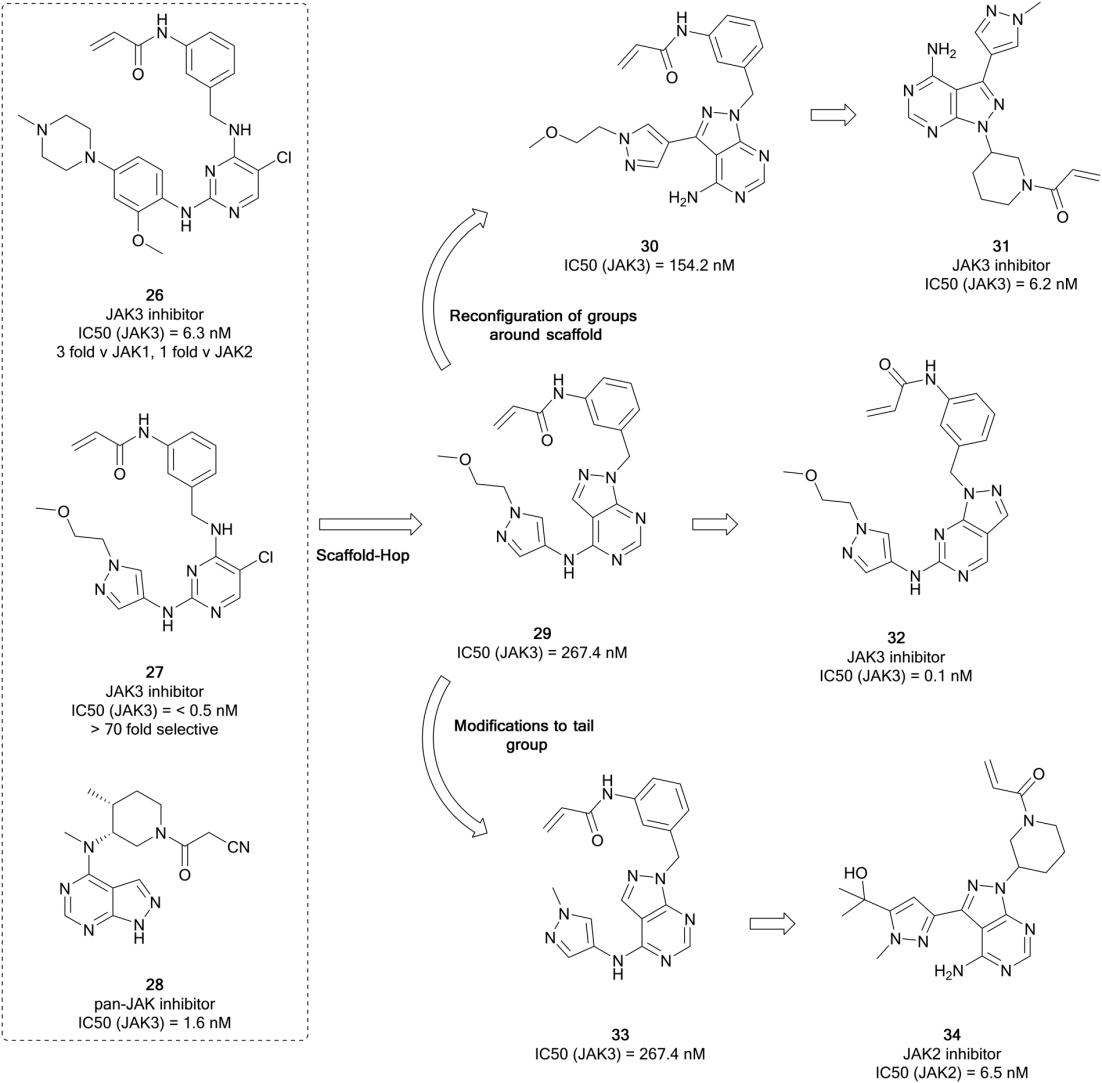
Development of selective JAK-family inhibitors from the parent molecule **26**.

The homology that exists between kinases with exposed cysteine residues has facilitated the design of JAK inhibitors such as **26** (latterly known as JAK-3-IN-1, Fig. 9) from covalent EGFR inhibitors, and it was identified as a moderately-selective JAK3 inhibitor, with limited potency *versus* EGFR. Further optimisation of this compound yielded **27**, a covalent JAK3 inhibitor, which had an improved IC_50_ value of <0.5 nM, 70-fold more potent than for EGFR.^76^ Yin *et al.* used these molecules as well as existing JAK-inhibitor SAR to replace the central pyrimidine ring system of **27**, with a bioisosteric pyrazolo[3,4-*d*]pyrimidine ring system in a scaffold-hop, giving parent compound **29** (Fig. 9), which showed a moderate JAK3 activity. Through molecular reconfiguration transferring the tail region to the C3-position of a 4-aminopyrazolopyrimidine core, **30**, the JAK3 potency was improved 2-fold from **29**. Using this structure and that of **3**, further optimisation of the alkyl chain of the tail and the linker to the Michael acceptor was achieved through several rounds of synthesis. Lead molecule **31** was identified with a JAK3 IC_50_ value of 6.2 nM, which was comparable to **26**. Furthermore **31** demonstrated unique selectivity over the other JAKs and also BTK and in *in vitro* studies it showed excellent antiproliferative activity in T cell cancer lines.^77^

Using moderately potent JAK3 pyrazolopyrimidine **29** as a starting point (Fig. 9), further work was carried out to develop more selective JAK3 inhibitors. Through rearrangement of the functional groups, in which the orientation of the pyrazolopyrimidine was shifted, moving the ‘tail-group’ to the C6-position as opposed to the C4-position, a novel library was obtained and studied further in a medicinal chemistry campaign in which the nature of the tail and the Michael acceptor groups were varied, according to literature examples. From this study, the most potent molecule was the direct rearrangement of **29** into **32**. **32** was highly potent and selective for JAK3 (IC_50_ = 1.5 nM and >376 fold selectivity over the other JAK isoforms) and further evaluation showed that it is selective for JAK3 over other kinases featuring a reactive cysteine group, such as the TEC family, EGFR and BTK. The lead molecules were evaluated for activity against T-cell cancer cells and were shown to display low-micromolar antiproliferative activity.^78^

Currently there are several JAK2 inhibitors in clinical use, ruxolitinib, baricitinib and fedratinib, with further examples in clinical trials. Yin *et al.* detailed the development of selective JAK2 inhibitors with the pyrazolo[3,4-*d*]pyrimidine scaffold, derived from the structure of **26**, as in their previous work, and aimed to exploit an acrylamide electrophile to form a covalent bond with a cysteine residue in the ATP site of JAK3. Mimicking the strategy to identify **31**, alterations to the tail region in the C4-position yielded **33** (Fig. 9), with no improvement in JAK3 potency. To develop more effective JAK3 inhibitors, a series of 1*H*-pyrazolo[3,4-*d*]pyrimidin-4-amine analogues were synthesised exploring the pyrazole ring, which also included a relocation of this heterocyclic group from the amino group at C4 to the C3 position of the pyrazolo[3,4-*d*]pyrimidin-4-amine. The compound library exhibited moderate JAK2 and JAK3 inhibitory activity with selectivity over JAK1. A further library was designed, and this resulted in reductions in the JAK3 potency and the enhancement of the JAK2 potency. This identified lead compound **34** that had a JAK2 IC_50_ value of 6.5 nM and >36-fold selectivity over the other JAK kinases.^79^

### MKK7

Mitogen-activated protein kinase 7 (MKK7) is a regulator of the c-Jun NH_2_-terminal protein kinase (JNK), a member of the MAPK family that regulates cell proliferation, differentiation, and apoptosis. MKK7 is a sensor of cellular stress, particularly associated with onogenesis.^80^ While MKK7 is implicated in T-cell acute lymphoblastic leukaemia, myeloma and lung cancers, its functions are context dependent and can be involved in cancer progression but also act as a tumour suppressor in many instances.^80^,^81^ Consequently, the development of MKK7 inhibitors has not been extensively studied, and no MKK7-inhibitors have entered clinical trials. Predominantly interest in these inhibitors is as probes to fully elucidate MKK7 function. MKK7 contains a cysteine residue, so covalent inhibition of the kinase has been proposed. However, the cysteine is highly conserved, so covalent inhibition is not as effective. Furthermore, achieving selectivity for MKK7 is challenging given significant homology with MAP2Ks.^80^

**3** and some derivatives display off-target MKK7 activity and has been used as a starting point for the development of MKK7 inhibitors. **23**, a dual EGFR/MKK7 inhibitor, was used by Wolle *et al.* to design and synthesise a small focussed library of pyrazolo[3,4-*d*]pyrimidines to exploit the homology between the binding-pockets of the EGFR and MKK7 active sites (Fig. 4). Through modifications and optimisation of the C3-position, *para*-ketobenzene **35** was identified as a lead molecule, with an IC_50_ value against MKK7 of 10 nM. Further evaluation of this molecule in a panel of 320 kinases demonstrated excellent selectivity with only seven other kinases possessing inhibition greater than 50%, predominantly those which possess a reactive cysteine residue.^82^ The library of MKK7 inhibitors containing Michael accepting groups were further studied, and the crystal structures solved to determine their binding modes. The most potent of the molecules studied contained the acrylamide group in **3**, with lead molecule **36** containing this group as its warhead and a phenol in the C3-position. Notably, **36** exhibited a greater MKK7 potency than **3** (IC_50_ = 8.6 nM).^83^

### mTOR

The mammalian target of rapamycin (mTOR) is a serine/threonine protein kinase that operates as the catalytic subunit of two protein complexes, mTORC1 and mTORC2, that act as sensors to integrate multiple extracellular and intracellular signals from the to coordinate the cell cycle.^84^,^85^ While increased mTOR expression and/or activation is observed in several cancers,^85^,^86^ the kinase itself is rarely directly mutated, with overactivation being triggered by mutations in upstream regulators, such as EGFR, PI3Ks, AKT, PTEN, RAS and RAF. Given its role as master regulator of many oncogenic signals, clinical evidence of mTOR activity has been found in over half of all cancers, making it an attractive target.^87^ There are two main classes of ATP competitive mTOR inhibitors: the dual mTOR/PI3K inhibitors, which arise as a result of significant similarities between these kinases, and selective mTOR inhibitors, which are more selective but unlike the rapalogs can inhibit the function of both mTOR complexexes. Both classes of ATP-competitive inhibitor have generated molecules entering clinical trials, with dactolisib and sapanisertib (**4**) examples of each respectively.^86^,^87^ The selective mTOR inhibitors, of which **4** is a leading example, have been frequently designed around the pyrazolopyrimidine core, such as a series of molecules designed by Intellikine.^88^,^89^

**Fig. 10:**
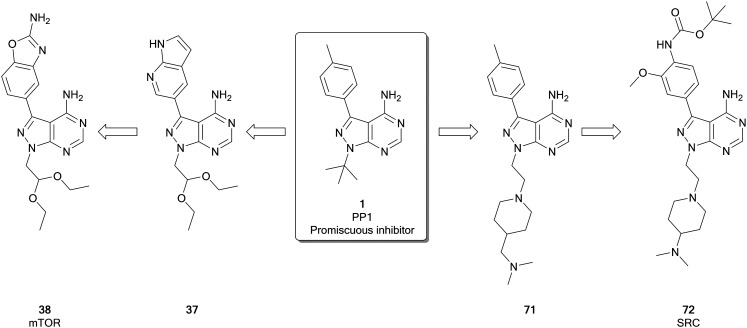
Development of potent and selective inhibitors of mTOR and SFK from the promiscuous kinase inhibitor **1**.

Fraser *et al.* recently identified a highly selective mTOR inhibitor, eCF309 (**38**), from a chemocentric phenotypic drug discovery approach focussed on the pyrazolo[3,4-*d*]pyrimidine scaffold. Hit molecule **37** (Fig. 10), first identified in a phenotypic screen, exhibited an IC_50_ (mTOR) of 328 nM. Optimisation of the C3- and N1-positions, incorporating different aryl moieties into the C3-position and substituted alkyl chains into the N1-position, yielded three lead molecules with low nanomolar potencies in breast and prostate cancer cell lines. The lead molecules all contained the characteristic benzo[*d*]oxazole group of **4** and were shown to induce cell cycle arrest in the cancer cell lines used. The kinome selectivity was determined, and **38** was identified as the most potent against mTOR (IC_50_ = 15 nM), as well as being highly selective *versus* other kinases. Further optimisation to the molecule's N1-position was attempted to explore potency and selectivity. While compounds with superior potency than **4** were identified, they displayed dual inhibition of mTOR and PI3Ks. The structural similarities between these dual inhibitors and **4** shed doubts over the supposed selectivity of **4** over PI3Ks. **38**, which features an ethyl acetal group at the N1-position of the ring, remained the most selective lead of the series.^25^ Subsequent investigations with an in-house library of 100 pyrazolo[3,4-*d*]pyrimidine derivatives, found the same family of compounds as promising antiproliferative leads against glioblastoma cell lines, including patient derived cells.^29^

### PAK

The p-21 activated kinases (PAKs) are a group of six serine/threonine kinases responsible for the regulation of numerous signalling pathways involved in proliferation, survival and motility, amongst others.^90^,^91^ While they are rarely directly mutated in cancer, the PAK kinases are subject to dysregulated expression and amplification, leading to uncontrolled cell proliferation, altered cell signalling, drug resistance and reduced levels of immune response through the activation of downstream signalling pathways, such as the PI3K/AKT pathway.^92^ Therefore, PAK inhibitors have been extensively studied and several inhibitors have reached early phase clinical trials. PF-3758309, a group II PAK4 inhibitor, was the first to reach clinical trials. However, it failed due to poor pharmacokinetic properties. The allosteric inhibitor KPT-9274, also a PAK4 inhibitor, is currently under evaluation in clinical trials for solid malignancies.^93^

**Fig. 11:**
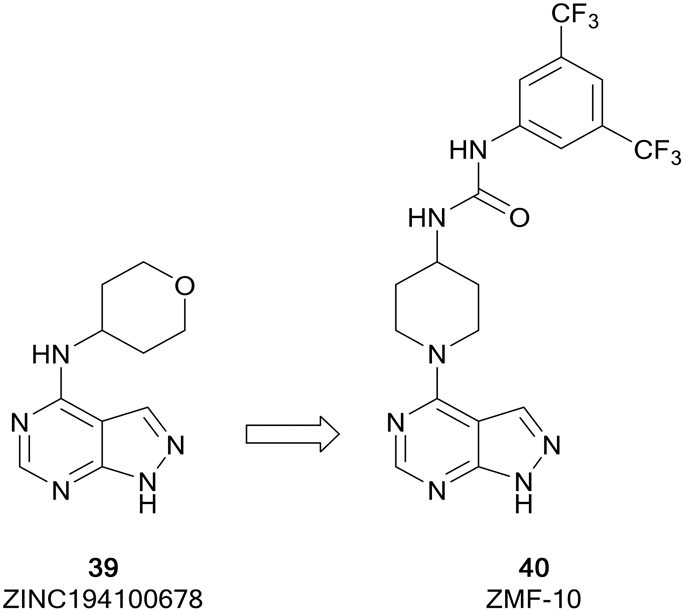
Design of PAK inhibitors through the modification of a virtual screening hit.

ZMF-10 (**40**) was identified as a sub-micromolar PAK1 inhibitor (Fig. 11), possessing moderate selectivity over other PAKs. **40** demonstrated an antiproliferative effect in breast cancer cell lines. The molecule was developed from ZINC194100678 (**39**), a hit identified through a virtual high-throughput screening (HTS) campaign, through docking of the molecules into a PAK1 homology model. A hydrophobic pocket identified by these studies aided the design of a series of molecules exploiting this region through modifications at the C4-position of the pyrazolo[3,4-*d*]pyrimidine scaffold. Following several rounds of optimisation, the lead molecule **40** was identified, which was confirmed as a PAK1 inhibitor and suppressed the proliferation of 4 cancer cell lines through the induction of apoptosis.^94^

### PI3K

The phosphatinidyl-3-kinases (PI3Ks), of which there are several isoforms (α, β, γ and δ), are responsible for the conversion of PIP2 to PIP3, which then initiates a complex network of downstream signalling.^95^ The α/β-isoforms are widely expressed, whereas the δ/γ-isoforms are predominantly expressed in B- and T-cells. Mutations in PI3Ks are observed in several different cancers, including glioblastoma, breast, ovarian, bladder, amongst others.^95^–^97^ The key challenge to the development of isoform selective PI3K inhibitors is that PI3Ks share homologous ATP-binding pockets resulting in non-specific pan-PI3K inhibitors, which display off-target effects. Idelalisib, approved in 2014 for the treatment of chronic lymphoblastic leukaemia, was the first PI3K inhibitor to reach the market.^97^

**Fig. 12:**
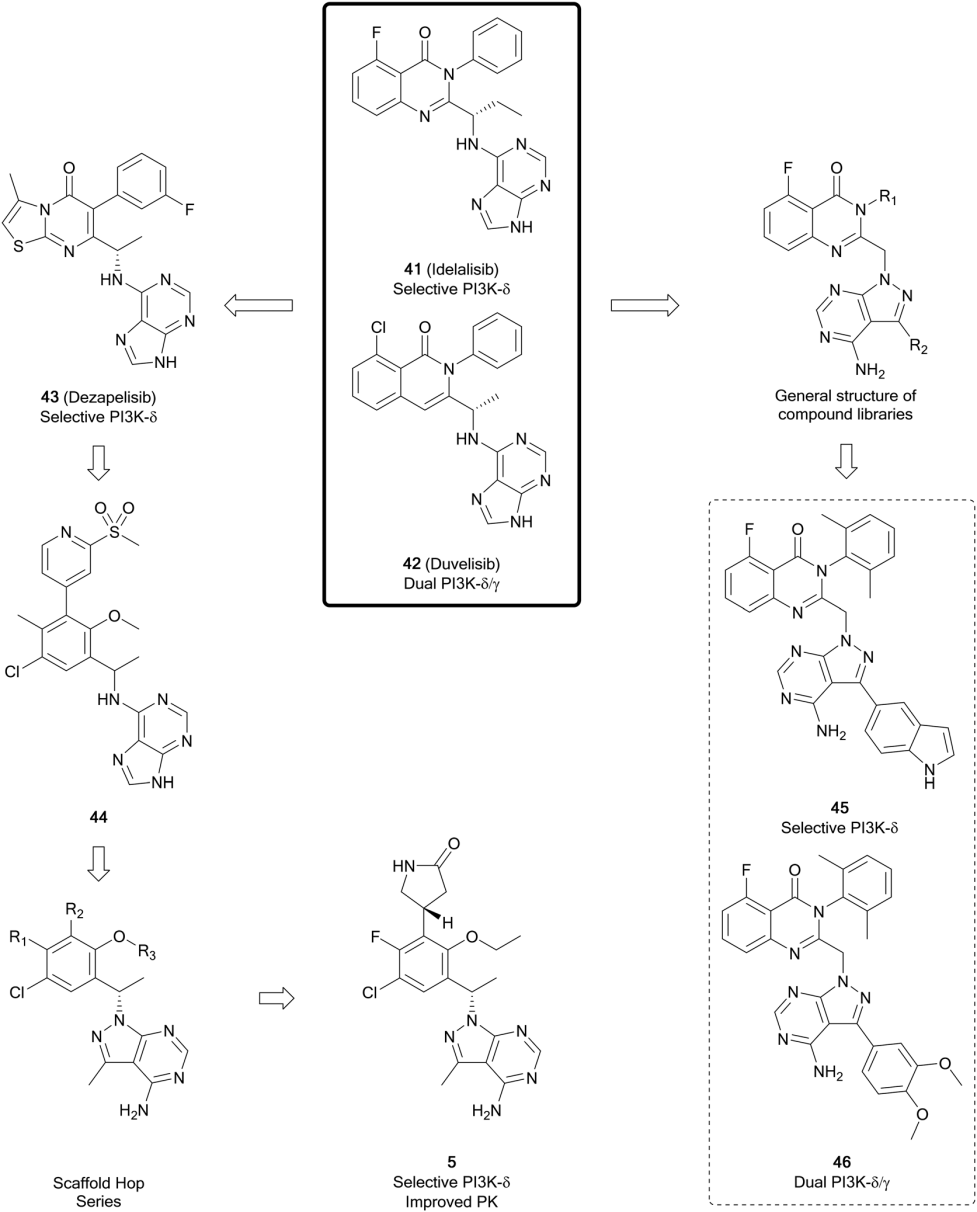
Development of PI3K inhibitors based on PI3Kδ-inhibitors **41** and **42**, yielding selective PI3K-δ inhibitor **5** and dual PI3K-δ/γ inhibitors.

The pyrazolo[3,4-*d*]pyrimidine core is found in several recent PI3K inhibitors, designed as derivatives of the purine scaffold of the selective PI3Kδ-inhibitor idelalisib (**41**) and the dual PI3Kδ/γ-inhibitor duvelisib (**42**). Parsaclisib (**5**), a pyrazolo[3,4-*d*]pyrimidine inhibitor currently being evaluated in clinical trials, was designed to improve the selectivity and PK properties of dezapelisib (**43**), a purine-containing derivative of **41** and **42** (Fig. 12). Through a systematic SAR study of **43**, purine lead **44** was identified. The central purine core of **44** was broken down and reassembled into a pyrazolo[3,4-*d*]pyrimidine ring system, whilst conserving its binding interactions. Initially there were promising improvements to the selectivity for the δ-isoform and the potency *versus* existing inhibitors. Further alterations to substituents around the pyrazolopyrimidine core removed hERG channel inhibition liabilities and resulted in the discovery of **5**, which exhibited efficacious reductions in the growth of tumours and low preclinical toxicity.^31^

In a series of novel quinazolinone compounds which demonstrated potent PI3K-δ inhibitory activity, Ma *et al.* incorporated the pyrazolopyrimidine core as a replacement for amine-linked purine cores in the approved inhibitors. The most potent PI3Kδ inhibitors, **45** and **46** (Fig. 12), were evaluated for their selectivity against other isoforms of PI3Ks, with **45** being selective for the δ-isoform (40–3630 fold) and **46** being a selective dual inhibitor of δ/γ over the other isoforms (820–1400 fold). The compounds demonstrated potent antiproliferative effects in a B-cell leukaemia cell line. Evaluation of PK properties identified that **46** was most suitable for further development due to its low clearance and good bioavailablity.^98^

**Fig. 13:**
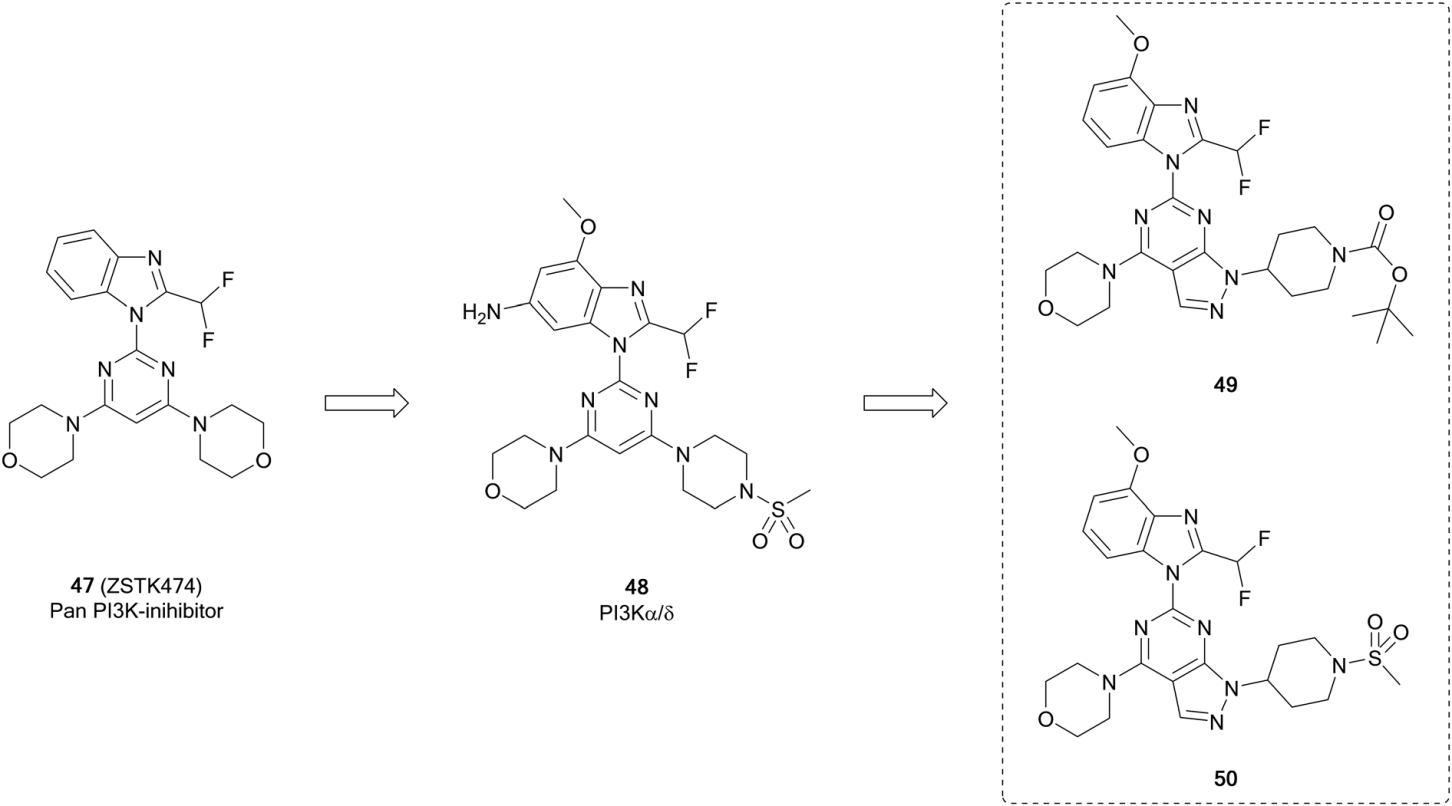
Design of selective PI3K-α inhibitor from pan-PI3K inhibitor **47**.

ZSTK474 (**47**, Fig. 13) is a further example of a potent ATP-competitive pan-PI3K inhibitor selective over other protein kinases, currently undergoing early phase clinical trials. Through modelling of **47**, Gamage *et al.* identified one of the two morpholines was not required for maintenance of the binding interactions and used this region for further optimisation through alterations to this position. Piperazinyl sulphonamide **48** is a potent dual inhibitor of PI3K-α/δ. The central pyrimidine ring of this structure was further optimised to a pyrazolo[3,4-*d*]pyrimidine, as well as to some purines. From the pyrazolopyrimidine derivatives synthesised, compounds **49** and **50** (Fig. 13) were shown to be potent and selective against specific isoforms of PI3K. **50** showed an IC_50_ value of 2.6 nM against PI3Kα with selectivity over other isoforms. Despite this high potency and selectivity profile, the purine analogues synthesised were more tractable for further development. However, the high potency of **49** and **50** demonstrates the effectiveness of the pyrazolopyrimidine scaffold to make PI3K inhibitors, serving as tool compounds.^99^

### RAF

The RAF kinases (ARAF, BRAF and CRAF) are important components of the MAPK/ERK signalling pathway. This pathway is one of the most frequently mutated in cancer, and is triggered through the activation of RAS, which in turn activates RAF. BRAF is the most frequently altered protein downstream of RAF, activated in 7–10% of all cancers.^100^,^101^ BRAF mutations increase MEK/ERK activity, promoting tumour proliferation and growth. 90% of the BRAF mutations comprise of the specific BRAF^V600E^ point mutation, which is a contributor to uncontrolled proliferation and angiogenesis.^102^ Sorafenib (**51**), a protein kinase inhibitor with activity against VEGFR, PDGFR and RAF kinases and approved for the treatment of kidney and liver cancers, was initially developed for inhibition of RAF. It exhibits selectivity for CRAF over BRAF. However, its lack of selectivity over other kinases has resulted in a low safe dose and consequently a loss of efficacy in some patients. More recent examples of RAF inhibitors include vemurafenib and dabrafenib, and these inhibitors are far more specific and effective, especially in melanomas which are driven by the BRAF-mutant BRAF^V600E^.^102^

**Fig. 14:**
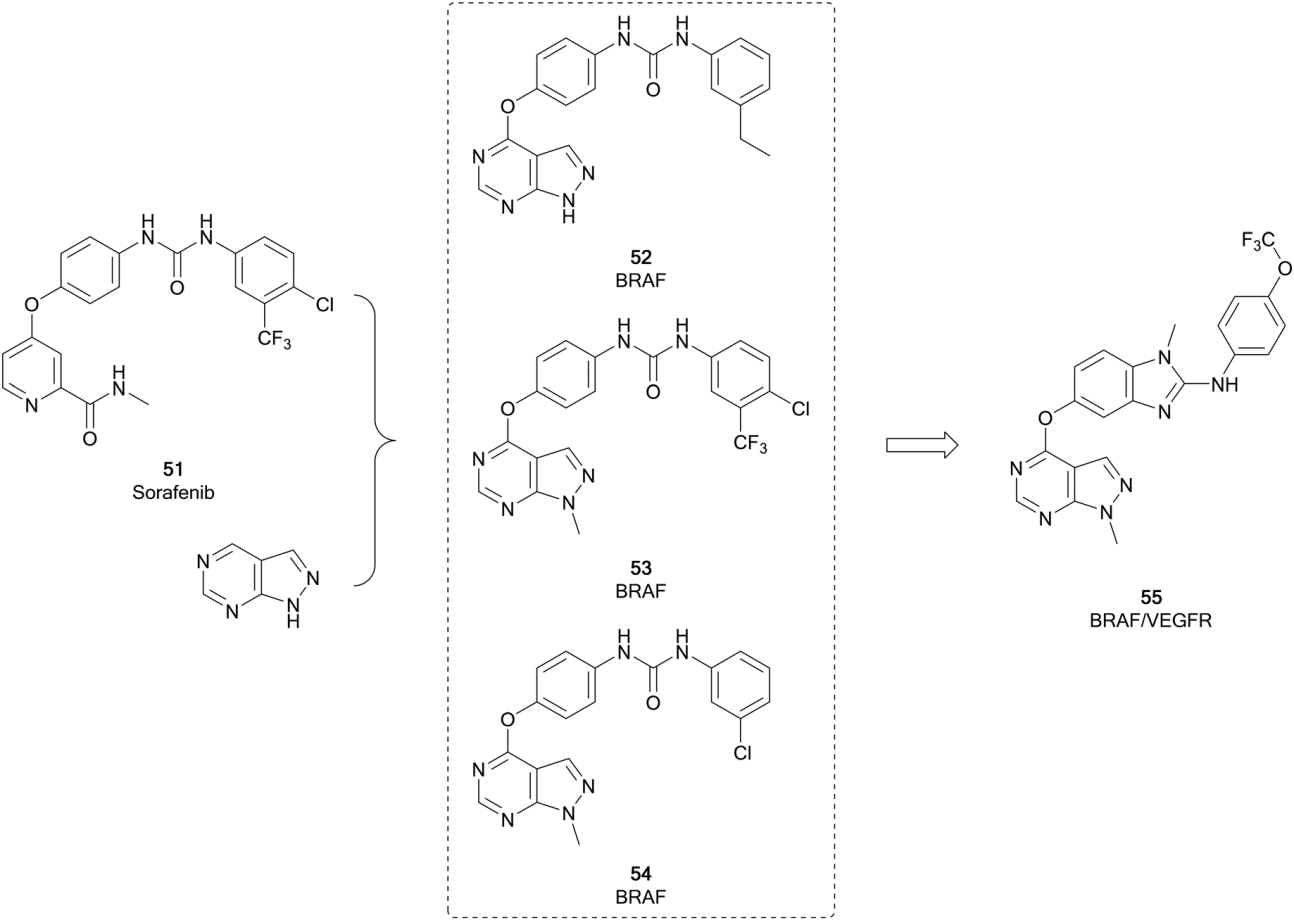
Development of BRAF kinase inhibitors derived from **51**. Subsequent optimisation yielded a dual BRAF/VEGFR inhibitor.

Fu *et al.* used **51** to design analogues, incorporating a pyrazolo[3,4-*d*]pyrimidine in place of the pyridine (Fig. 14). The substitution pattern on the distal aromatic ring, linked to the core through the urea, was altered to explore the scaffold SAR. The N1-position was also explored, through the addition of some substituted alkyl groups. Three lead compounds, **52**, **53** and **54**, were identified, exhibiting potent BRAF^V600E^ inhibition. The antiproliferative effect of the leads were evaluated in human cancer cell lines, with **53** being more effective than **51**. Notably, **53** was also more potent than **51***versus* BRAF^V600E^ at 23.6 nM. **53** was further evaluated against other kinases, confirming potent inhibitory activity against other RAF isoforms, but no significant activity against other kinases tested, indicating **53** is a potent and selective pan-RAF inhibitor.^103^ The inhibition of RAF shows a degree of synergy with inhibition of VEGFR, and it has been proposed that inhibition of RAF, which contributes to tumour growth, coupled with inhibition of VEGFR, which contributes to angiogenesis (providing the tumour with nutrients), would be an effective way to treat some cancers.^104^**53** has been subject to further optimisation, to develop a dual BRAF/VEGFR inhibitor through improving the low VEGFR inhibition while maintaining RAF inhibition. Modelling of **53** into the crystal structure of VEGFR indicated good overlap of the pyrazolopyrimidine into the ATP pocket, with the urea region being required to enhance binding. The rigidity of the urea region was enhanced by scaffold hopping with an aminobenzoxazole or an aminobenzimidazole ring, which linked to a distal aromatic ring (Fig. 14). The target compound library, which varied the substitution of the distal ring system, was screened against BRAF^V600E^ and VEGFR2 with **51** as a positive control. Several compounds showed high inhibition of both kinases, comparable to the control. **55** showed sub-micromolar potencies against both kinases (0.171 and 0.779 μM respectively), as well as effective antiproliferative properties in several cancer cell lines. The kinome selectivity of **55** showed little to no inhibition of a range of kinases even at the highest concentration, except CRAF. **55** was later shown to be a cell cycle arrestor in HUVEC cells.^105^

**Fig. 15:**
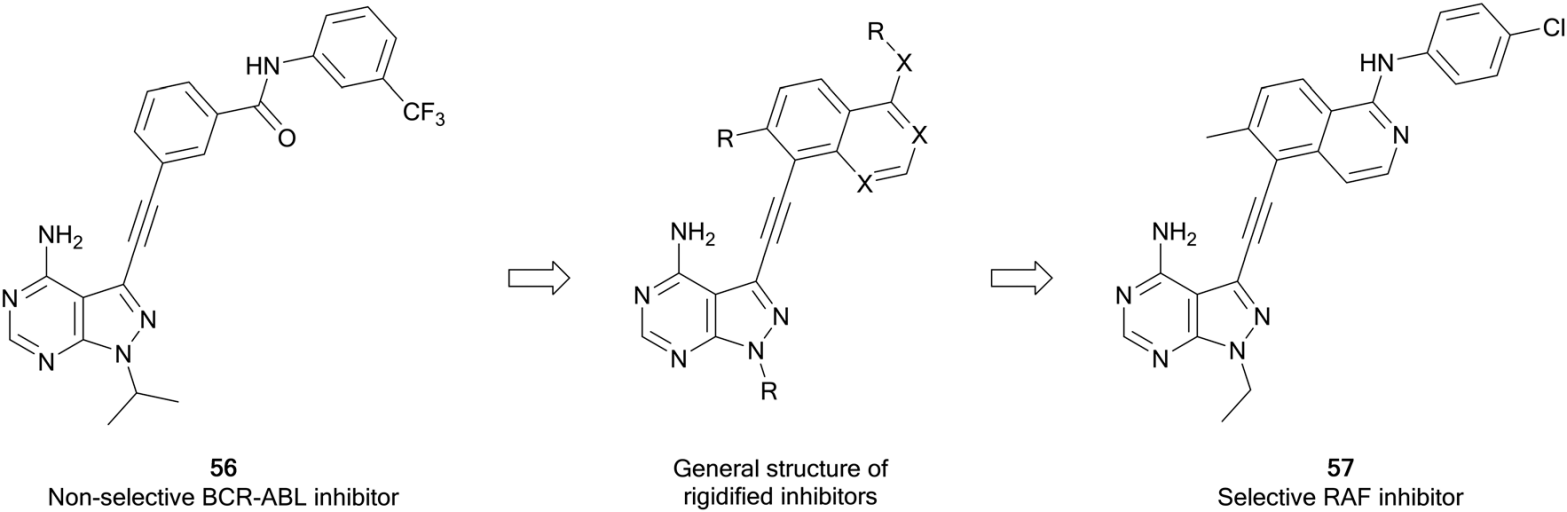
Design of selective RAF inhibitors from promiscuous inhibitors through a strategy of rigidification.

Zhang *et al.* have recently developed a new series of pyrazolo[3,4-*d*]pyrimidine inhibitors that possess excellent selectivity for RAF kinases. Using an existing library of promiscuous 3-alkynyl-pyrazolo[3,4-*d*]pyrimidine BRC-ABL inhibitors, such as **56**, a strategy of rigidification was used to restrict the inhibitory profile to more flexible kinases (Fig. 15). Alkyne-linked naphthyl, isoquinoline and quinazoline pyrazolopyrimidines were designed from **56**, synthesised and screened for activity against BRAF and growth inhibition. The quinazolines were the most potent, with **57** exhibiting the best potency against BRAF^V600E^, with an IC_50_ value of 8 nM. The kinase selectivity profile of **57** against 245 kinases showed only four kinases including BRAF and CRAF were inhibited by over 85%. Evaluation in BRAF^V600E^ mutant cancer cells indicates that **57** can be potentially effective against BRAF mutant cancers.^106^

### RET

RET is a receptor tyrosine kinase activated by a family of extracellular signalling molecules known as the GDNFs. Binding to its ligands homodimerises RET, which in turn activates downstream signalling pathways such as MAPK and PI3K/AKT. Overexpression and activation of RET are present in the pathogenesis of many human cancers, particularly thyroid, lung and some brain cancers.^107^,^108^ In 2019, the FDA approved selpercatinib as the first selective RET-inhibitor for the treatment of thyroid cancer and non-small cell lung carcinoma (NSCLC). Prior to this, two multi-kinase inhibitors that targeted RET had been approved for use in thyroid cancer, cabozantinib (**20**) and vandetanib. However, these inhibitors possess off-target effects against VEGFR and other kinases, leading to undesirable side-effects in the clinic.^109^

**Fig. 16:**
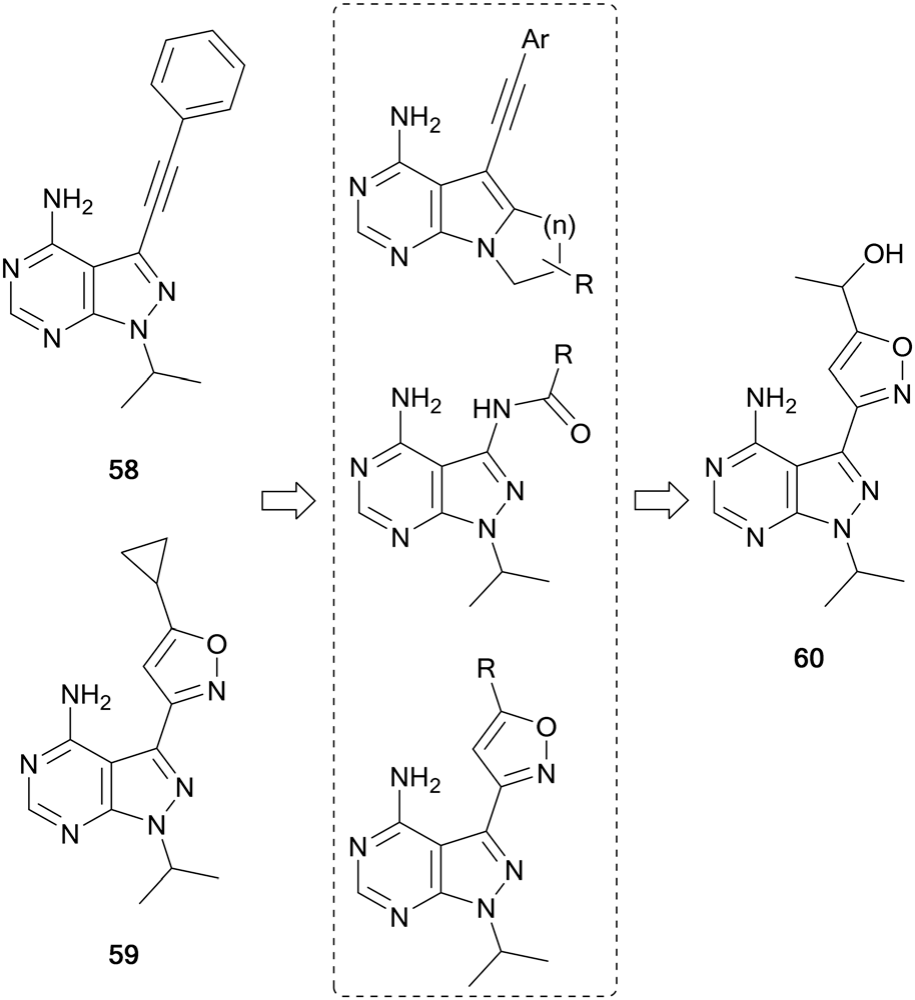
Design and synthesis of pyrazolo[3,4-*d*]pyrimidine RET inhibitors.

The first pyrazolo[3,4-*d*]pyrimidine kinase inhibitor, **1**, has been shown to possess activity against RET.^110^ Wang *et al.* identified two further pyrazolopyrimidines, **58** and **59** (Fig. 16), with nanomolar potency against RET and high selectivity against VEGFR2. However, this potency was not demonstrated in cellular assays. The leads were optimised through the design of three compound libraries, introducing modifications through ring-closing, addition of new functional groups and linkers and the addition of amides to exploit a hydrophobic pocket. The compounds were screened for RET inhibition, and the most potent series were the isoxazole ring analogues. These analogues explored the functional groups appended to this ring and the most potent was **60**, whose IC_50_ value was 61 nM. Studies of **60** identified that it was selective for RET over VEGFR2, and also inhibited phosphorylation of STAT3 and AKT, downstream effectors of RET signalling.^111^

### SFK and ABL

The SRC-family kinases (SFK) comprises a group of nine non-receptor tyrosine kinases: SRC, YES, FYN, FGR, LCK, HCK, BLK, LYN and FRK. This enzyme family plays a key role in the transduction of a wide range of extracellular signals, *via* receptor tyrosine kinases, cell-to-cell and extracellular matrix-to-cell communications. While this family of kinases is not frequently mutated, it is central for the transmission of many oncogenic signals, promoting survival, angiogenesis, proliferation and invasion.^112^ Activation of SRC itself is observed in half of all cancers, making it an attractive target for cancer therapy.^112^,^113^ Numerous SFK inhibitors have been evaluated for the treatment of cancers in clinical trials. Dasatinib was the first approved inhibitor of SRC, although because of its promiscuity it also inhibits many other kinases, including ABL. Abelson tyrosine kinases 1 and 2 (a.k.a. ABL and ARG, respectively) are implicated in processes of cellular differentiation, division, adhesion, and stress response. In chronic myeloid leukaemia (CML), a chromosomal translocation occurs to generate a new fusion gene, *BCR-ABL*, which is transcribed into the constitutively active fusion protein BCR-ABL,^114^ being the main driver of this disease. Dasatinib was approved for the treatment of several forms of leukaemia, including CML, due to its capacity to inhibit *BCR-ABL*-positive cancers, and it is undergoing evaluation for use in lymphomas, breast, prostate and other cancers in clinical trials. Further dual SRC/ABL inhibitors have since been evaluated in clinical trials and gained approval, including bosutinib.^15^,^115^ However, none have yet been approved for their direct treatment of SRC-driven cancers, probably due to their promiscuity.

**Fig. 17:**
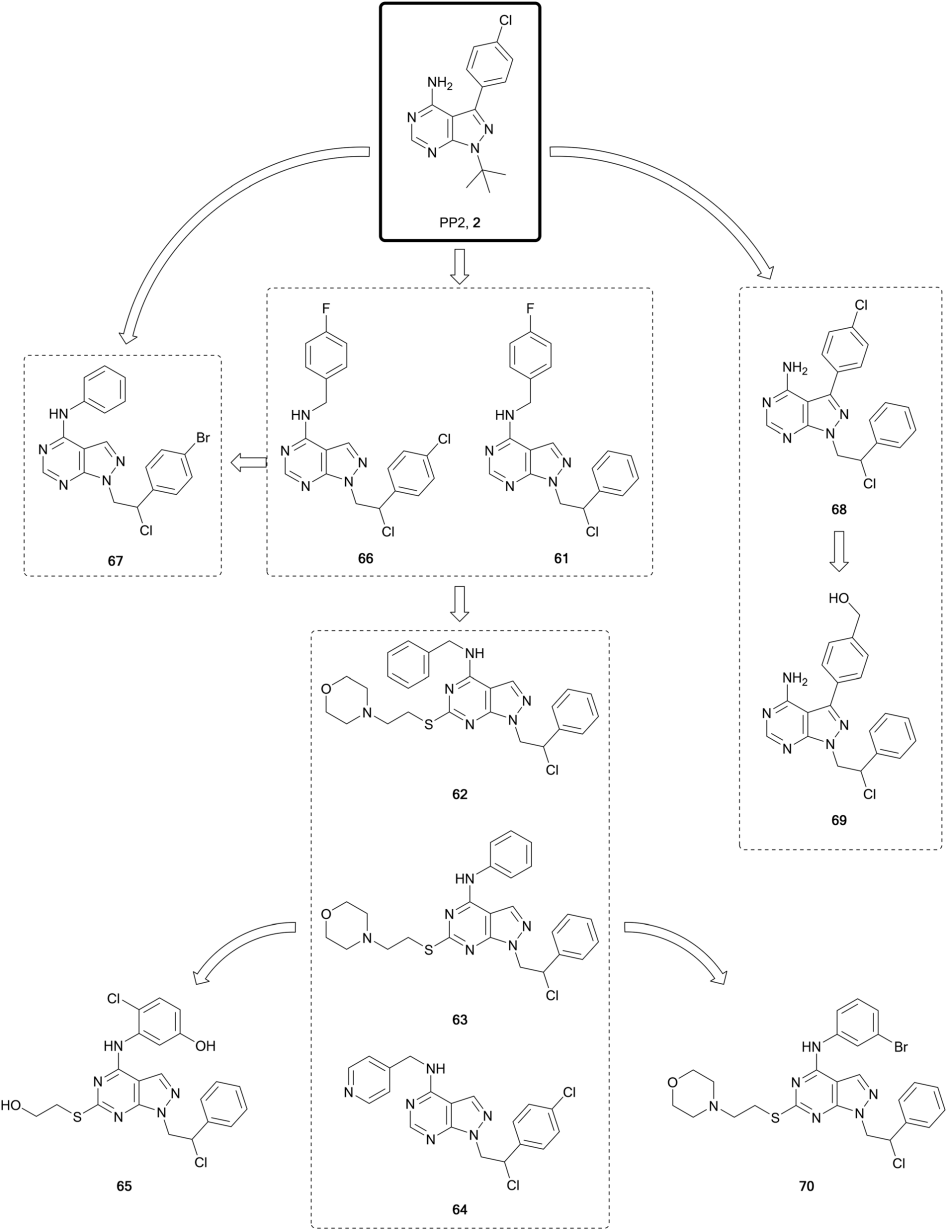
Design and evolution of inhibitors of SFK and ABL from the promiscuous inhibitor **2**.

Studies conducted by the Schenone Lab, initially based on the promiscuous inhibitors **1** and **2**, resulted in the generation of a large library of pyrazolo[3,4-*d*]pyrimidines, many of which have been published (Fig. 17). In 2008 study, they disclosed a dual pyrazolo[3,4-*d*]pyrimidine inhibitor of SRC and ABL, **61**.^116^ A later lead optimisation campaign sought to improve the physicochemical properties of **61**, while maintaining its dual inhibitory activity. Modelling identified that different polar moieties could be incorporated in the C4- and C6-positions of the scaffold, which were solvent exposed, thereby improving the water solubility and ADME properties. The compound library synthesised largely retained the dual SRC/ABL inhibitory profile upon biological evaluation. The lead compounds were investigated for antiproliferative properties in a panel of leukaemia cell lines. Further evaluation of the most promising compounds was carried out under hypoxic conditions, facilitating the identification of the most promising leads; **62**, **63** and **64** (Fig. 17), as optimised analogues of **61**, with improved ADME properties and favourable inhibitory properties.^117^ The crystal structure of the compound **63** bound to SRC was used to design modifications to the amine-linked benzene in the C4-position. In parallel to these optimisations, alterations were made to the side chain in the C6-position. The compound library was screened *in vitro* to determine SRC inhibitory activity and cellular viability in neuroblastoma cells. The lead compound from the screens, **65**, demonstrated greater potency than the starting compound in neuroblastoma cells.^118^

One of the challenges in finding selectivity between ABL and SRC is the significant levels of homology that exist between them. As a result, screening of the pyrazolopyrimidine library against ABL *in silico* identified several hits, such as **66**, another derivative of **60** from the 2008 study, which was used to design a small focussed library of ABL inhibitors. The lead molecule from this series, **67**, demonstrated nanomolar potencies against ABL, SRC and the BCR-ABL mutant T315I, which is one of the mutants responsible of the acquired resistance to the 1st generation BCR-ABL inhibitor imatinib. A dual inhibitor of BCR-ABL and its mutant T315I would provide a better standard of treatment. *In vivo* studies of **67** in mice inoculated with 32D-T315I cells showed that those treated showed a significant reduction >50% in tumour size with respect to the placebo.^119^

For the treatment of heterogeneous lymphomas and other B-cell cancers, it is proposed that inhibition of multiple targets could provide a greater therapeutic benefit for patients, due to complex mutational pathways present in these cancers. In B- and T-cell tumours mutations in FYN, BLK and LYN can coexist, and it was postulated that an inhibitor of these three targets would demonstrate greater efficacy. **68** (Fig. 17), a FYN inhibitor first identified in 2015, was used as the starting point for the development of a multitarget inhibitor. The compound was modelled into the protein structures to identify areas that could be exploited for co-inhibition of the kinases. The phenyl ring in the C3-position of the pyrazolo[3,4-*d*]pyrimidine core was selected for modification given its position in the inner side of the ATP binding pocket. Some of the 12 synthesised derivatives also examined the chlorine group in the N1-sidechain. A phenotypic screen against different cancer cell lines (B-cells and T-cells) identified the lead **69** with low micromolar potencies across all the cell lines. Kinome screening demonstrated that the compound was a multitarget inhibitor of FYN-BLK-LYN with comparable potencies across the three kinases.^120^

ATP-competitive SRC inhibitor **70** (Fig. 17), a derivative of **63**, has been studied as a potential treatment for glioblastoma, both *in vitro* and *in vivo*, demonstrating good growth inhibition effects, while also being well tolerated and having low off-target toxicity.^121^**70** has been evaluated in a series of invasive patient-derived glioblastoma cell lines, two from the invasive region and a further obtained from the core region of the tumour. The compound demonstrated low micromolar IC_50_ values (7–11 μM) against the three cell lines studied.^122^ A prodrug strategy was used to improve the solubility of 9 compounds from the pyrazolopyrimidine library, including **70**, through addition of a water-solubilising group containing a *N*-methyl piperazine. The prodrug of **70** was the most efficient and demonstrated a comparable efficacy to **70***in vivo* in an orthotopic model of glioblastoma multiforme (GBM).^123^

Using ligand-based inhibitor design and phenotypic screening cooperatively and starting from the promiscuous inhibitor **1**, a series of pyrazolo[3,4-*d*]pyrimidines were synthesized and tested against breast cancer cells by Fraser *et al.* (Fig. 10). After several iterations consisting of design, synthesis and phenotypic screening of focussed libraries (up to 12 compounds per round), a family of highly selective SRC inhibitors was identified. The chemical strategy primed the incorporation of flexibly linked solubilising groups to the N1-position of the pyrazolopyrimidine scaffold, aiming to enhance the physicochemical properties of **1** and to explore SAR within a chemical space with freedom to operate. Structural evolution was based on anticancer potency against ER+ and triple negative breast cancer cells, thus discriminating compounds with low cell permeability and inhibitors of pathways not involved in these cancer subtypes. Hit molecule **71** was identified for further optimisation of the *p*-tolyl group in the C3-position, since this position is key for the determination of kinase selectivity. Further rounds of optimisation inspired in the works of Schenone and others, resulted in the discovery of the lead inhibitor eCF506 (**72**). Further optimisation attempts resulted in a wide range of compounds with different activities, but negligible or no improvement of potency. **72** was further evaluated for kinase selectivity, demonstrating high SRC potency (IC_50_ = <0.5 nM) and >950-fold selectivity over ABL and other kinases. Given its excellent physicochemical properties and promising *in vitro* DMPK profile, **72** was further evaluated in cancer models and *in vivo* (zebrafish and mice).^113^ Mirroring the activity of ABL/nonSRC inhibitor imatinib, **72** is the only *in vivo* active SRC inhibitor with selectivity over ABL.

### TAM

The TAM family of kinases, comprising of TYRO3, AXL and MER, are a group of receptor tyrosine kinases that are amplified in an array of solid and haematological malignancies. Amplification or overactivation of TAM kinases triggers downstream signalling associated with uncontrolled proliferation and survival.^124^ In cancer, TAM kinases are able to supress tumour immunity, as well as contributing to drug resistance. AXL is the most frequently amplified of the TAM kinases.^125^ The FDA has approved some multi-kinase inhibitors, such as cabozantinib (**20**), which has moderate AXL activity. Further compounds are under preclinical and clinical evaluation for a variety of cancers, including several compounds designed specifically for TAM kinases.^66^,^126^

**Fig. 18:**
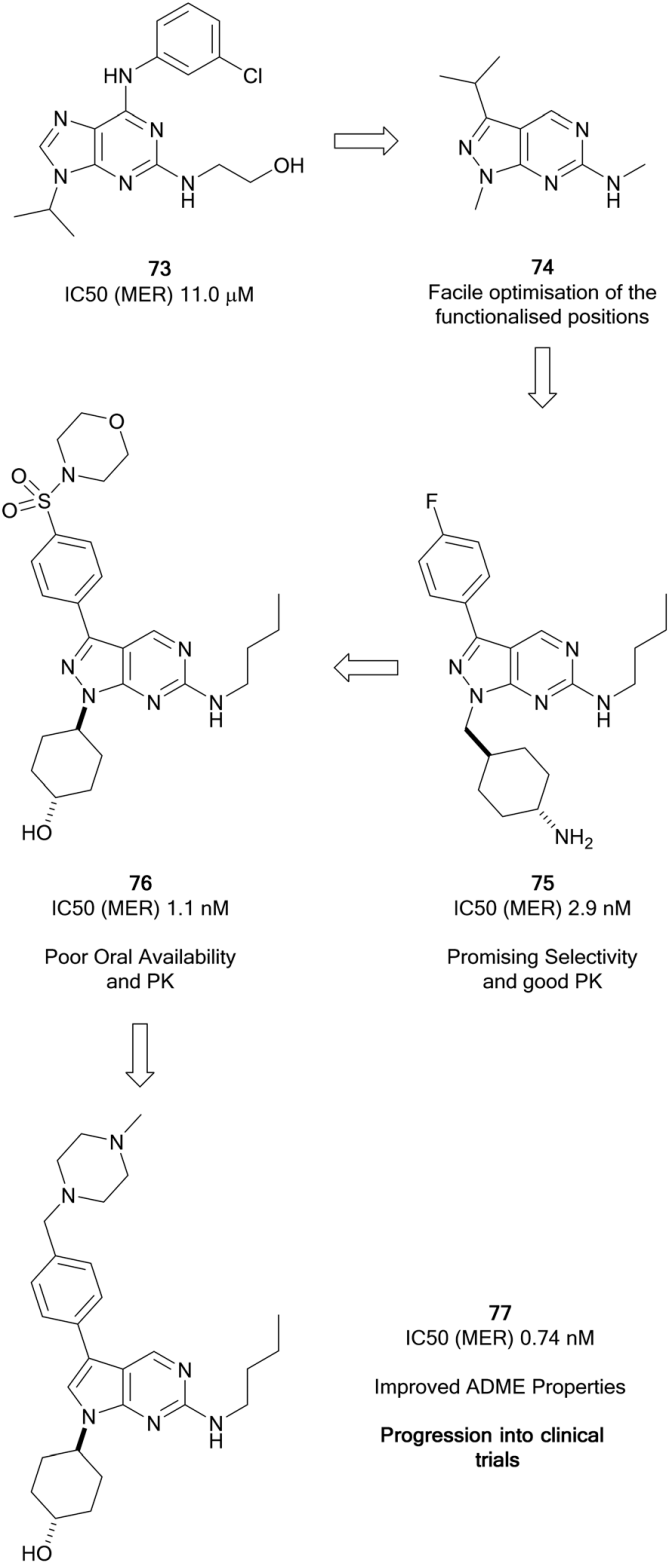
Development of Mer kinase inhibitors by the Wang group leading to clinical candidate **77**.

Wang *et al.* focussed on the development of inhibitors of kinases of the TAM family starting from a weak MER inhibitor, **73** (Fig. 18), yielding numerous promising inhibitors for the treatment of various forms of leukaemia, one of which has progressed to clinical trials.^127^ Through use of a targeted-discovery approach, derivative **74**, a pyrazolo[3,4-*d*]pyrimidine heterocycle was proposed, and structural modelling indicated that modifications to three independent sites, the N1-, C3- and C6-positions would enable optimisation of the scaffold for the inhibition of MER. Optimisations of these positions yielded UNC569 (**75**), which had good potency and selectivity for MER, while also maintaining advantageous DMPK properties.^128^ Undesirable activity towards the hERG ion channel made **75** unsuitable for further clinical development given the risk of cardiotoxicity associated to this inhibition. The primary amine of the appending group in the N1-position was postulated to contribute to off-target hERG activity. Alteration to the groups here tuned out the hERG inhibition and further optimisation to the C3-position through introduction of a sulphonamide yielded the inhibitor UNC1062 (**76**), which had improved selectivity and potency, but a poor PK profile.^129^ Through work to improve the PK profile of **76** by lowering of the lipophilicity of the molecule and reduction of molecular weight through removal of the morpholine and subsequently the sulphonamide, as well as removal of a nitrogen from the pyrazolopyrimidine core, UNC2025 (**77**) was discovered (Fig. 18). **77** possessed a greatly improved PK profile while maintaining the potency, and was subject to a scale-up and further *in vivo* investigations which demonstrated effective inhibition.^130^**77** progressed to a clinical trial and demonstrated effectiveness in acute myeloid leukaemia patients.^127^

**Fig. 19:**
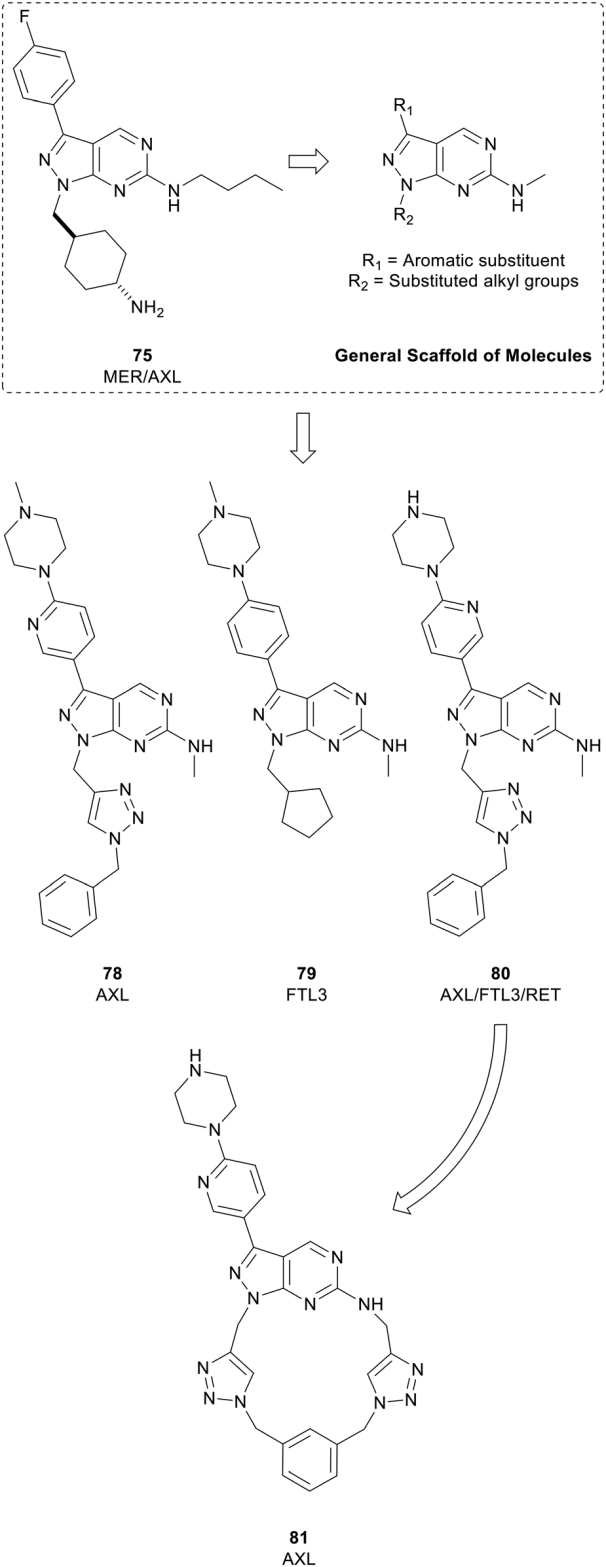
Development of kinase inhibitors for AXL kinase. Lead compound **80** (eSM119) was optimised for AXL-selectivity through a strategy of macrocyclization to give **81** (eOC148).

Myers *et al.* used a chemocentric phenotypic drug discovery approach to develop a series of molecules based on **75**, a compound reported by Wang *et al.* that possessed similar potency against MER and AXL. By modifying the groups in the N1- and C3-positions with substituted alkyl groups and aromatic substituents respectively, a library of 6-methylaminopyrazolo[3,4-*d*]pyrimidines were generated (Fig. 19). Screening against AXL-expressing and non-expressing cancer cells was used to bias compound selection and evolution towards AXL inhibition. Lead molecule eSM134 (**78**, IC_50_ = 380 nM) is of note as it inhibits AXL with great selectivity over other TAM kinases, and other RTKs. A further molecule of interest from this library was the FTL3 inhibitor eSM156 (**79**), which possessed an IC_50_ value of 1.4 nM *versus* FTL3, and sub-micromolar potencies against leukaemia cell lines.^131^ eSM119 (**80**) also demonstrated promising activity against three oncogenic kinases (AXL/FTL3/RET) with sub-micromolar potencies. **80** was optimised for AXL-selectivity through a strategy of macrocyclization (Fig. 19). Macrocycles of 12 atoms or more may demonstrate medicinal properties, often because of constrained rotational degrees of freedom. Loratinib is an example of a recently approved macrocyclic kinase inhibitor. Exploitation of the triazole moiety of **80** yielded a macrocyclic analogue containing two triazoles, eOC148 (**81**) that was synthesised using a double copper-catalysed azide-alkyne cycloaddition reaction. **81** was screened for activity against TAM family members and RTKs FTL3 and RET based on the profile of **80**. While the screen showed macrocycle **81** was a moderate inhibitor of AXL (IC_50_ = 13 μM), it did not inhibit the other members of the TAM family or RET and had over 4-fold selectivity over FTL3, a significant shift from **80**. Furthermore, **81** displayed potent antiproliferative activity against MV4-11 cells, a myeloid leukaemia cell line dependent on AXL for survival.^132^

### VEGFR

Vascular endothelial growth factor receptor (VEGFR) is a family of three transmembrane receptor tyrosine kinases that bind growth factors extracellularly, which triggers homodimerization, cross-phosphorylation of their intracellular kinase domains and subsequent phosphorylation of multiple substrates.^133^ VEGFR plays a key role in angiogenesis, the formation of new blood vessels, as well as controlling immune cells present in the microenvironment. Tumours initiate the process of angiogenesis through VEGFs to provide them with nutrients, allowing them to grow.^134^ Inhibition of these receptors has been used to starve tumour environments of nutrients and potentially limit their growth. Sorafenib (**51**) was the first VEGFR inhibitor approved by the FDA for use in renal cell carcinoma. **51** is a multi-kinase inhibitor, and has numerous other targets, a common characteristic of VEGFR inhibitors due to its homology of the ATP-site with other kinases. Further multi-kinase inhibitors for VEGFR have also been approved and developed for the treatment of cancer, including sunitinib, pazopanib, vandetanib and cabozantinib (**20**).^135^

**Fig. 20:**
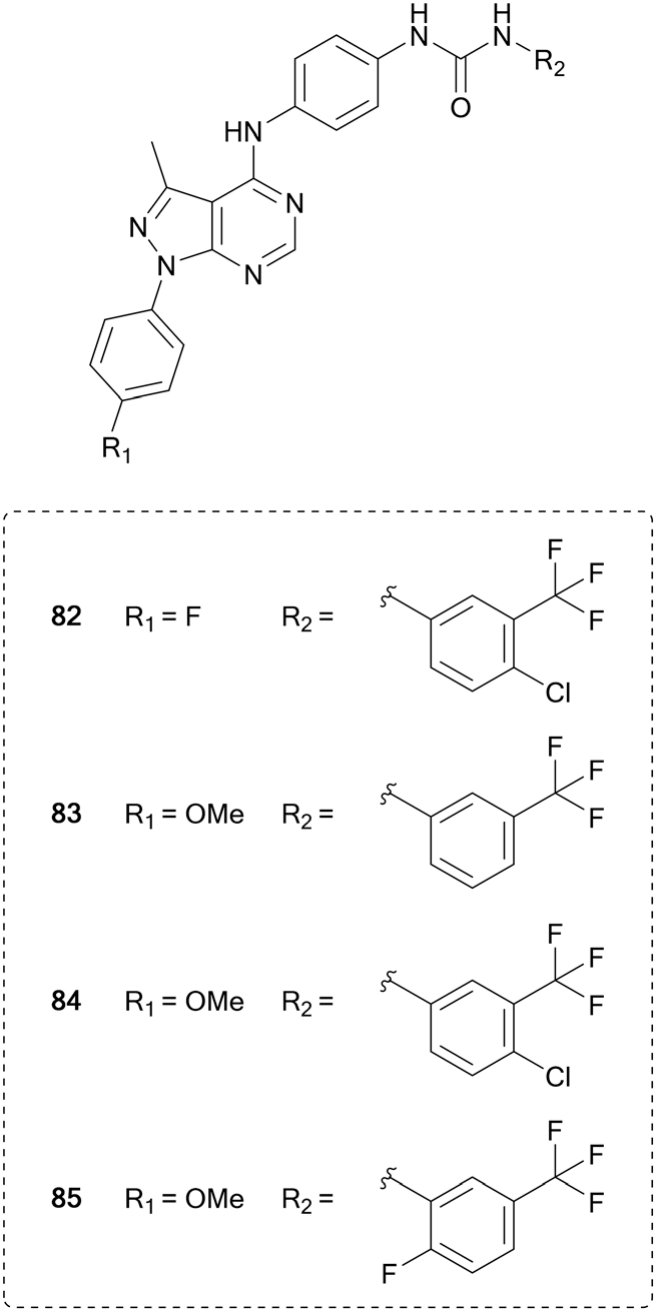
Development of kinase inhibitors for VEGFR kinase from sorafenib by modification of the distal aromatic ring.

Pyrazolo[3,4-*d*]pyrimidines have been designed to inhibit this VEGFR2, based on the different binding modes of sorafenib (**51**) and linifanib, which binds to the ATP pocket or to an allosteric pocket of VEGFR, respectively. Maintaining the urea linked aryl-binder of the allosteric site, Kassab *et al.* replaced the fused ring ATP-binder with a pyrazolo[3,4-*d*]pyrimidine and designed 16 compounds primarily featuring variations at the distal urea linked phenyl ring and minor modifications to the N1-phenyl ring. The compounds were evaluated in a panel of 60 tumour cell lines. Several compounds showed broad anticancer activity, and the four lead molecules (**82–85**, Fig. 20) were evaluated for their VEGFR inhibition, demonstrating submicromolar IC_50_ values. **84**, the most potent hit (IC_50_ = 220 nM), displayed comparable inhibitory effects of VEGFR than **51**.^136^

## Conclusion and future directions

As shown in this review, the pyrazolo[3,4-*d*]pyrimidine scaffold is a powerful starting point in medicinal chemistry programmes due to facile derivatisation with a wide range of different groups and functionalities. The independent or combined modification of the moieties at the N1-, C3-, C4- and C6-positions have been the focus of much research to modulate the kinase selectivity of this privileged scaffold to specific or multiple targets. Its bioisosterism with adenine, which confers the ability to bind to the hinge region of the ATP site of many kinases even more potently than adenine, makes this scaffold highly applicable in the development of new kinase inhibitors. The exploitation of this scaffold has been particularly fruitful for the development of kinase inhibitors for the treatment of cancer. The fact that one pyrazolo[3,4-*d*]pyrimidine, the BTK inhibitor ibrutinib (7th best-selling cancer drug of 2018), has been approved to treat B-cell cancers and several more inhibitors targeting different kinases are in advanced clinical trials, demonstrate both the therapeutic potential and versatility of this scaffold.^137^ In the coming years, it is highly likely that further pyrazolo[3,4-*d*]pyrimidines will be approved for the treatment of cancer patients, especially for indications where there is limited or no targeted therapies available. And given the broad bioactive chemical space around this core yet to be uncovered, there is little doubt that medicinal chemists will continue investing efforts to thoroughly explore the possibilities that the pyrazolo[3,4-*d*]pyrimidine scaffold are yet to offer.

## Conflicts of interest

There are no conflicts to declare.
